# A novel group of negative-sense RNA viruses associated with epizootics in managed and free-ranging freshwater turtles in Florida, USA

**DOI:** 10.1371/journal.ppat.1010258

**Published:** 2022-03-11

**Authors:** Thomas B. Waltzek, Brian A. Stacy, Robert J. Ossiboff, Nicole I. Stacy, William A. Fraser, Annie Yan, Shipra Mohan, Eugene V. Koonin, Yuri I. Wolf, Thais C. S. Rodrigues, Pedro H. O. Viadanna, Kuttichantran Subramaniam, Vsevolod L. Popov, Veronica Guzman-Vargas, Lisa A. Shender

**Affiliations:** 1 Department of Infectious Diseases and Immunology, College of Veterinary Medicine, University of Florida, Gainesville, Florida, United States of America; 2 NOAA, National Marine Fisheries Service, Office of Protected Resources, University of Florida (duty station), Gainesville, Florida, United States of America; 3 Department of Comparative, Diagnostic, and Population Medicine, College of Veterinary Medicine, University of Florida, Gainesville, Florida, United States of America; 4 Florida Department of Agriculture and Consumer Services, Bronson Animal Disease Diagnostic Laboratory, Kissimmee, Florida, United States of America; 5 National Center for Biotechnology Information, National Library of Medicine, National Institutes of Health, Bethesda, Maryland, United States of America; 6 Center for Biodefense and Emerging Infectious Diseases, Institute for Human Infections and Immunity, the University of Texas Medical Branch, Galveston, Texas, United States of America; 7 Fish and Wildlife Research Institute, Florida Fish and Wildlife Conservation Commission, Gainesville, Florida, United States of America; Emory University School of Medicine, UNITED STATES

## Abstract

Few aquatic animal negative-sense RNA viruses have been characterized, and their role in disease is poorly understood. Here, we describe a virus isolated from diseased freshwater turtles from a Florida farm in 2007 and from an ongoing epizootic among free-ranging populations of Florida softshell turtles (*Apalone ferox*), Florida red-bellied cooters (*Pseudemys nelsoni*), and peninsula cooters (*Pseudemys peninsularis*). Affected turtles presented with similar neurological signs, oral and genital ulceration, and secondary microbial infections. Microscopic lesions were most severe in the softshell turtles and included heterophilic/histiocytic meningoencephalitis, multi-organ vasculitis, and cytologic observation of leukocytic intracytoplasmic inclusions. The virus was isolated using Terrapene heart (TH-1) cells. Ultrastructurally, viral particles were round to pleomorphic and acquired an envelope with prominent surface projections by budding from the cell membrane. Viral genomes were sequenced from cDNA libraries of two nearly identical isolates and determined to be bi-segmented, with an ambisense coding arrangement. The larger segment encodes a predicted RNA-directed RNA polymerase (RdRP) and a putative zinc-binding matrix protein. The smaller segment encodes a putative nucleoprotein and an envelope glycoprotein precursor (GPC). Thus, the genome organization of this turtle virus resembles that of arenaviruses. Phylogenetic analysis shows that the RdRP of the turtle virus is highly diverged from the RdRPs of all known negative-sense RNA viruses and forms a deep branch within the phylum Negarnaviricota, that is not affiliated with any known group of viruses, even at the class level. In contrast, the GPC protein of the turtle virus is confidently affiliated with homologs from a distinct group of fish hantaviruses. Thus, the turtle virus is expected to become the founder of a new taxon of negative-sense RNA viruses, at least with a family rank, but likely, an order or even a class. These viruses probably evolved either by reassortment or by intrasegment recombination between a virus from a distinct branch of negarnaviruses distant from all known groups and a hanta-like aquatic virus. We suggest the provisional name Tosoviridae for the putative new family, with Turtle fraservirus 1 (TFV1) as the type species within the genus Fraservirus. A conventional RT-PCR assay, targeting the TFV1 RdRP, confirmed the presence of viral RNA in multiple tissues and exudates from diseased turtles. The systemic nature of the TFV1 infection was further supported by labeling of cells within lesions using *in situ* hybridization targeting the RNA of the TFV1 RdRP.

## Author summary

Freshwater turtles are an important part of aquatic ecosystems, but relatively little is known about diseases that affect them. We discovered a new virus associated with die-offs involving multiple turtle species in Florida. Affected turtles had inflammation involving the nervous system and other organs indicative of viral infection. We isolated a virus from diseased turtles and characterized its morphology using electron microscopy. We also sequenced its entire genome to explore its relatedness to other viruses and developed a PCR-based detection assay. The morphology of the virus particle and genome characteristics were reminiscent of an arenavirus. However, phylogenetic analyses of two viral proteins indicate that the turtle virus most likely evolved through genetic mixing between a distinct branch of RNA viruses distant from all known groups and an aquatic hantavirus. Thus, the turtle virus is expected to become a founding member of a new RNA virus family. We provide evidence linking this novel virus with disease in both wild and captive turtles.

## Introduction

Florida has the longest coastline in the contiguous United States (US) comprised of nationally significant estuaries and the only barrier coral reef in North America. These vital marine ecosystems are intimately tied to the landscape by lakes, swamps, and winding aquifer fed rivers. Thus, Florida’s iconic aquatic wildlife, economy, and heritage are inextricably linked to the health of its diverse aquatic ecosystems. Among Florida’s notable aquatic wildlife are chelonians (turtles, tortoises, and terrapins) that occur in varied terrestrial, freshwater, and coastal ecosystems. Fifty-seven chelonian species occur in the US comprising nearly a fifth of all known species in the world, and of these, more than half occur in Florida [1,2]. River drainages of the southeastern (SE) US that flow into the Gulf of Mexico, including the Escambia and Apalachicola drainages that meander through Florida, possess high chelonian species richness that rivals the most diverse region in the country, the Mobile River Basin [1,2,3]. The chelonian diversity in the SE US is dominated by freshwater aquatic to semi-aquatic turtle families including the Emydidae (cooters, sliders, map turtles, painted turtles, and box turtles), Trionychidae (softshell turtles), Kinosternidae (mud and musk turtles), and Chelydridae (snapping turtles). Importantly, only a few of the many turtles in the SE US are included in global conservation strategies, and thus, the region has been identified as a global priority area for chelonian conservation [3].

Chelonians represent one of the most imperiled groups of vertebrates, with around 10% of species listed as critically endangered by the IUCN Red List of Threatened Species [4,5]. Furthermore, approximately two-thirds of the assessed turtle species are considered threatened [4,5]. Chelonians have low fecundity, low juvenile survival rates, and long adult life spans, thus the loss of mature animals has a significant impact on population recovery. Even slight increases in adult mortality rates are thought to be enough to lead to population declines [6] and can require prolonged periods for recovery [7]. Although the primary threats to non-marine chelonians are believed to be habitat loss and over-collection associated with food and international trade markets [4], increasingly recognized are the negative impacts of emerging infectious diseases including viruses [8,9,10].

Viruses of ectothermic vertebrates, including chelonians, have received far less attention than viruses that infect endothermic vertebrates and impact human and agricultural health. However, over the past few decades, the number of genetically characterized chelonian viruses has increased through PCR amplification coupled with Sanger sequencing and more recently via next-generation sequencing technologies. A number of DNA virus families have been characterized from reptiles including members of the Orders *incertae sedis* (*Anelloviridae*), *Rowavirales* (*Adenoviridae*), *Chitovirales* (*Poxviridae*), *Zurhausenvirales* (*Papillomaviridae*), *Cirlivirales* (*Circoviridae*), *Piccovirales* (*Parvoviridae*), *Pimascovirales* (*Iridoviridae*), and *Herpesvirales* (*Herpesviridae*) [11]. However, only herpesviruses and iridoviruses have been associated with impactful diseases in aquatic chelonians [10,11,12,13].

As with DNA viruses, a number of RNA virus families have also been characterized from reptiles including members of the Orders *Nidovirales* (*Arteriviridae*, *Tobaniviridae)*, *Martellivirales* (*Togaviridae*), *Amarillovirales* (*Flaviviridae*), *Ortervirales* (*Retroviridae*), *Picornavirales* (*Caliciviridae*, *Picornaviridae*), *Reovirales* (*Reoviridae*), *Mononegavirales* (*Bornaviridae*, *Paramyxoviridae*, *Rhabdoviridae*, *Suniviridae*), and *Bunyavirales* (*Arenaviridae*, *Hantaviridae*, *Nairoviridae*, *Peribunyaviridae*) [11]. The order *Bunyavirales* includes 12 viral families that possess enveloped nucleocapsids with multipartite (2–8 segments), single-stranded, negative-sense or ambisense RNA genomes [14,15]. Bunyaviruses include notable human (e.g., hantaviruses and Crimean-Congo hemorrhagic fever virus), veterinary (e.g., Rift Valley fever virus), and plant (e.g., tomato spotted wilt virus) pathogens. Many of these viruses are vectored by blood-sucking (e.g., mosquitoes or ticks) or sap-sucking (e.g., thrips) arthropods (arborviruses), and rodents (roboviruses). In recent years, bunyaviruses within the families *Hantaviridae*, *Nairoviridae*, and *Arenaviridae* have been characterized from squamate reptiles or the ticks that feed on them [Reviewed in 11]. However, the only bunyaviruses proven to be pathogenic in reptiles are arenaviruses (genera *Reptarenavirus* and *Hartmanivirus*) that induce inclusion body disease in boid and phythonid snakes.

In this work, we characterized a virus isolated from diseased Florida freshwater turtles from a farm and from an ongoing epizootic among wild populations. The moribund free-ranging freshwater turtles presented with similar clinical signs, gross lesions, and microscopic lesions. The growth of these turtle viruses in cell culture facilitated their downstream ultrastructural and genomic characterization. Genomic sequencing of the turtle viruses enabled comparative genomic and phylogenetic analyses that revealed the phylogenetic affinities of their individual genes. These analyses indicate that the turtle virus, which we provisionally denote Turtle fraservirus 1 (TFV1), likely evolved by genome segment reassortment or by intrasegment recombination, with the large segment coming from a distinct branch of negarnaviruses distant from all known groups and the small segment or at least the gene encoding the virus glycoprotein precursor derived from a hanta-like aquatic virus. The TFV1 sequences were then used to develop specific molecular tools that confirmed the virus was present in multiple tissues and individuals of three freshwater turtle species collected during the epizootic.

## Methods and materials

### Ethics statement

Examination and analyses of all animals were conducted under University of Florida Animal Care and Use Committee (IACUC) protocol #202107642.

### Field investigation and animal examinations

**Fig 1:**
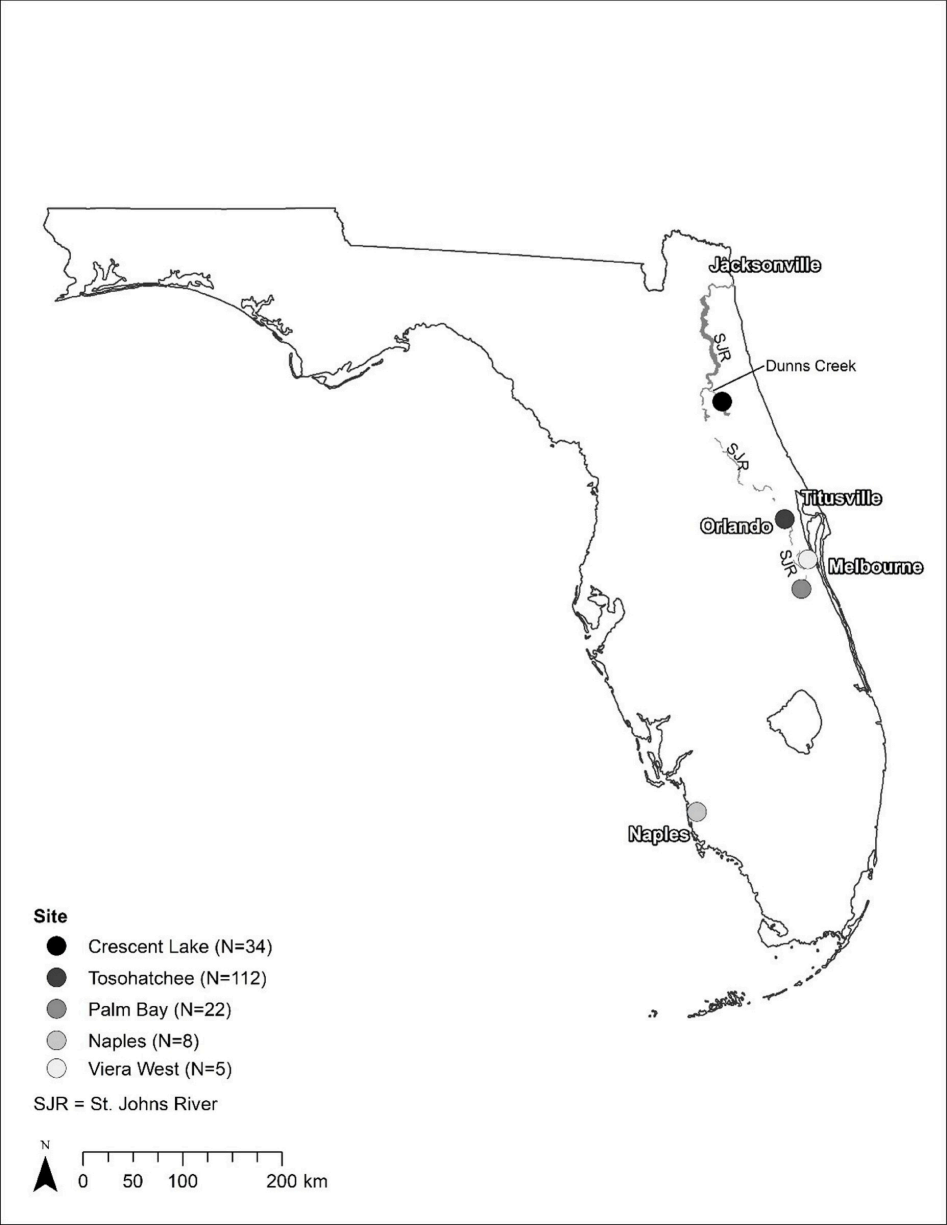
Locations of sampled free-ranging turtles from 13 March 2018 to 27 February 2019, and 23–24 March 2020. Numbers represent the estimated counts of sick and dead turtles reported from these regions, as detailed in Table 1.

Freshwater turtles were collected for evaluation from five discrete field sites (Table 1, Fig 1). The Florida Fish and Wildlife Conservation Commission (FWC) initially received reports of moribund and dead Florida softshell turtles (*Apalone ferox*) from field staff and members of the public near the Tosohatchee Wildlife Management Area (TWMA) along the St. Johns River (SJR) in March 2018. The SJR uniquely flows from south to north, and the upper basin receives water from a system of marshes, swamps, canals, and water management areas that comprise the SJR headwaters. The FWC performed eight airboat surveys near the TWMA to assess the magnitude of the mortality event and to collect moribund and dead turtles. Additional public reports (verified with photos when possible) were received from Crescent Lake, which connects to the SJR via Dunn’s Creek. A single mortality event, reported to FWC by local government officials, occurred in a system of retention ponds in an urban park in Naples. In early 2019, staff from the SJR Water Management District again reported finding morbid and dead freshwater turtles in the SJR headwaters. Following this report, FWC conducted two airboat surveys in the system of canals near Palm Bay. In March 2020, in response to public reports of sick and dead softshells, FWC did a site investigation at a cluster of interconnected stormwater drainage system ponds in the community of Viera West, located approximately 10 km to the east of the SJR.

**Table 1:** Sites from which sick and dead freshwater turtles were examined and tested positive for Turtle fraservirus 1 during period of 13 March 2018 through 25 February 2019, and March 2020.

Site #	Site Name	County	Latitude^1^	Longitude^1^	Date Range^2^	Total^3^
1	Tosohatchee	Brevard, Orange, and Seminole	28.563014	-80.941112	13 Mar to 23 May 2018	112
2	Naples	Collier	26.21623	-81.73389	25 Mar to 16 April 2018	8
3	Crescent Lake	Putnam	29.491752	-81.506062	29 April to 3 June 2018	34
4	Palm Bay	Brevard	28.009149	-80.79024	28 Jan, and, 25 Feb 2019	22
5	Viera West	Brevard	28.243006	-80.730152	23–24 Mar 2020	5

^1^Latitude and longitude values correspond to the coarse scale map locations as shown in Fig 1. Sites spanning large linear waterbodies (Tosohatchee and Palm Bay) are represented by the centroid of a minimum convex polygon placed around all GPS locations.

^2^Represents time period during which surveys were performed or mortality reports received.

^3^Total count of known or estimated sick and dead freshwater turtles recorded during the specified date range.

From all study sites, live and dead turtles were opportunistically collected for examination. Live, overtly debilitated and/or lethargic turtles were humanely euthanized using phenobarbital following heavy sedation with tiletamine and zolazepam. As controls, additional sick freshwater turtles were euthanized and necropsied from areas outside of the listed study sites. When possible, antemortem blood samples were collected from the jugular or coccygeal vein, or the subcarapacial sinus. Blood films were prepared and stained with Wright-Giemsa stain (Harleco, EMD Millipore) and reviewed for quantitative and morphological assessment of blood cells. Plasma chemistry was performed using a Hitachi 917 chemistry analyzer (Roche Diagnostics). Blood culture was performed using standard methods. Whole blood lead was analyzed using Magellan LeadCare (Meridian Bioscience).

Necropsies were completed using standard methods. Tissue imprints of liver and spleen were prepared and stained with Wright-Giemsa stain (Harleco, EMD Millipore) for cytological evaluation. Curved carapace length and weight were recorded. Nutritional condition was subjectively scored 1 (poor) to 4 (robust) based on condition of the body fat. Samples of most major organs, including the nervous system, and gross lesions were frozen at -80°C. Frozen samples of blood and urine and touch impressions of organs and lesions for cytological examination were collected from a subset of turtles.

Tissues for histopathology were fixed in 10% neutral phosphate buffered formalin, processed into paraffin blocks, sectioned onto glass slides, and stained with hematoxylin and eosin using routine methods. Selected tissues were also stained using Brown-Hopps, Ziehl-Neelsen acid fast, and/or Gomori methenamine-silver methods as needed to detect or characterize intralesional microorganisms. In 2019, histopathology for most cases was limited to the brain, spinal cord, liver, and spleen due to resource limitations. These tissues were selected based on observations in 2018 as a means of cost-effective screening for lesions that were characteristic of the previous mortality event.

Concurrent with the disease investigation involving free-ranging turtles, we examined an earlier epizootic event involving managed softshell turtles in Florida. A turtle farmer submitted a moribund softshell turtle of unknown species to the Bronson Animal Disease Laboratory in Kissimmee, FL for assistance with investigating a morbidity and mortality event at the facility in 2007. The animal was euthanized, and postmortem samples were collected for virus isolation as described below. This event was of interest because we strongly suspected that the free-ranging turtle epizootic was viral, initial PCR results for previously reported chelonian viruses, including ranaviruses, were negative, and there is a general paucity of information on diseases of trionychid turtles in the region.

### Virus isolation

Virus isolation was undertaken as part of investigations of both the farmed and free-ranging turtle epizootics. For the 2007 farmed softshell turtle epizootic, pooled samples of liver, kidney, and spleen were homogenized (10% w/v) in minimum essential medium (MEM) supplemented with 2 mM L-glutamine and 50 μg gentamicin/ml. A cell-free ultrafiltrate was prepared by centrifugation for 1 min at 3,000 x g in a 1.8-ml microcentrifuge tube, followed by filtration of the supernatant through a 0.45 μm filter. The ultrafiltrate was inoculated onto a confluent monolayer of Terrapene heart (TH-1) cells grown in a 25 cm^2^ tissue culture flask. After 1 h of adsorption, 10 ml of growth medium (MEM plus 10% fetal bovine serum and 50 μg gentamicin/ml) was added, and the cultures were incubated at room temperature (25°C) and observed daily for cytopathic effects (CPE).

On 24 March 2020, FWC personnel collected and euthanized a moribund Florida softshell turtle from the Viera West study site. The turtle was necropsied at FWC the same day, and samples (liver, kidney, spleen, brain, and urine) were delivered fresh to the UF Wildlife and Aquatic Veterinary Disease Laboratory (WAVDL) in Gainesville, FL. Each of the four tissues and the urine sample were diluted 1:25 in Leibovitz’s Media (L-15, GE Healthcare Life Sciences). The tissue samples were then homogenized at high speed using a stomacher (Seward Stomacher 80 Biomaster) for 120 s. The four homogenates and the urine sample were then centrifuged at 3000 x g (10 min at 4°C) to pellet cellular debris. An equal volume of L-15 containing 2% antibiotic-antimycotic (AA; Gibco) was added to the clarified tissue homogenates and urine sample. After incubating overnight at 4°C, the five samples were clarified once again at 3,000 x g (10 min at 4°C) and inoculated onto 25 cm^2^ flasks containing confluent monolayers of TH-1 cells. After a 1 h viral adsorption period, the inoculum was removed from each flask and replaced with L-15 containing 2% Fetal Bovine Serum (Gibco) and 1% AA. Flasks were incubated at room temperature (25°C) and observed daily for CPE. If CPE was observed on viral passage 1 (VP1), the supernatant was clarified as described above and used to inoculate monolayers of TH-1 cells that had reached confluency within the last 24 h. The VP2 cultures were observed daily for 14 days postinoculation (dpi).

### Transmission electron microscopy (negative staining)

Virus particles were partially purified from the supernatant of a TH-1 cell culture displaying extensive CPE following inoculation with the pooled internal tissue homogenate of the managed softshell turtle in 2007. Cell culture medium including cell debris was homogenized and centrifuged at 4,000 x g for 10 minutes. The supernatant from the first centrifugation was further centrifuged at 14,000 x g for 60 min. The resulting pellet was suspended in 25 μl deionized water, further diluted in water to reduce turbidity then mixed with an equal volume of 1.5% phosphotungstic acid (pH 6.8) and applied to grids covered with carbon-coated polyvinyl formal film using a micropipettor. After excess fluid was removed by touching the fluid with filter paper, the sample was air dried, and viewed on a ZEISS model EM109 electron microscope.

### Transmission electron microscopy (ultrathin sections)

The VP2 supernatant from the 25 cm^2^ flask of TH-1 cells, originally inoculated with the 2020 softshell turtle kidney homogenate, was decanted after extensive CPE was observed. The TH-1 cells were fixed in 5 ml of modified Karnovsky’s fixative (2P+2G, 2 % formaldehyde prepared from paraformaldehyde and 2 % glutaraldehyde in 0.1 M cacodylate buffer pH 7.4). After a 15 min fixation at room temperature, cells were scraped off the flask and centrifuged at 3,000 x g (10 min at 4°C). The resulting cell pellet was resuspended in phosphate-buffered saline (PBS) and shipped overnight on ice packs to the University of Texas Medical Branch Department of Pathology Electron Microscopy Laboratory (UTMB-EML). At UTMB-EML, the cell pellet was washed in cacodylate buffer and left in 2P+2G fixative overnight at 4°C. The next day, the cell pellet was washed twice in cacodylate buffer, post-fixed in 1 % OsO4 in 0.1 M cacodylate buffer pH 7.4, *en bloc* stained with 2 % aqueous uranyl acetate, dehydrated in ascending concentrations of ethanol, processed through propylene oxide, and embedded in Poly/Bed 812 epoxy plastic (Polysciences). Ultrathin sections were cut on a Leica ULTRACUT EM UC7 ultramicrotome (Leica Microsystems), stained with 0.4 % lead citrate, and viewed on a JEM-1400 electron microscope (JEOL USA) at 80 kV. Mean virion diameter was calculated from measurements analyzed in ImageJ2 [16].

### Genome sequencing and analysis

RNA was extracted from the TH-1 cell culture supernatants, from both the 2007 and 2020 cases, using a QIAamp Viral RNA Mini Kit (Qiagen) according to the manufacturer’s instructions. Separate cDNA libraries were generated using a NEBNext Ultra RNA Library Prep Kit (Illumina) and sequenced on an Illumina MiSeq sequencer. *De novo* assembly of paired-end reads was performed using SPAdes 3.10.1 with default parameters [17] and the assembled contigs were subjected to BLASTX searches in OmicsBox 1.2.4 (BioBam Bioinformatics) against the National Center for Biotechnology Information (NCBI) nonredundant protein database. The integrity of the assembled genome sequence was verified by mapping the reads to the consensus sequence and inspecting the alignment in CLC Genomics Workbench 10.1.1 (Qiagen), using a window size of 1 bp. Putative open reading frames (ORFs) were determined using GeneMarkS (http://exon.biology.gatech.edu/) [18] and protein function was predicted using BLASTP searches against the NCBI non-redundant protein sequence database. The signal peptide and transmembrane domains were predicted using the Simple Modular Architecture Research Tool (http://smart.embl.de/). The glycosylation sites were predicted using the NetNGlyc 1.0 Server (http://www.cbs.dtu.dk/services/NetNGlyc/). In addition, all assembled contigs from the 2007 and 2020 datasets were compared to each other using BLASTN analysis to rule out the existence of additional segments encoded by the TFV1. Contigs that showed >90% similarity were scrutinized further by comparing against the NCBI non-redundant nucleotide and protein databases using BLASTN and BLASTX analyses, respectively. Contigs that showed no significant similarities to the database were screened for coding sequences using the Expasy translate tool (https://web.expasy.org/translate/). The genome sequences of the 2007 and 2020 TFV1 isolates have been deposited in the NCBI GenBank database under accession nos. OM471797-OM471798 and MZ458542-MZ458543, respectively.

### Sequence comparison and phylogenetic analysis

For the sensitive detection of domains of predicted protein sequences, the amino acid (aa) sequences of TFV1 were compared to databases of protein family profiles using HHPred [19]. Additionally, TFV1 protein sequences were compared to a collection of custom profiles for the RdRPs of all known RNA viruses, in order to identify and classify the highly diverged TFV1 RdRP [20,21]. For the purpose of phylogenetic analysis, amino acid sequences of the negarnavirus RdRP and bunyavirus-like glycoprotein were aligned separately using MUSCLE5 [22]. Alignment regions, with more than 2/3 frequency of gap characters and homogeneity below 0.05 were removed [23]. Maximum likelihood phylogenetic analysis was performed using IQ-TREE with aBayes bootstrap support [24], under the rtREV+F+R9 and WAG+F+R9 evolutionary models for the RdRP and the glycoprotein, respectively, selected by the built-in model finder.

### Development of a reverse transcription PCR assay

A specific Turtle fraservirus 1 (TFV1) reverse transcription PCR (RT-PCR) assay was designed to amplify a 405 bp region within the coding sequence of the TFV1 RNA dependent RNA polymerase (L segment). The RT-PCR assay was used to screen the virus isolate resulting from cell cultures displaying CPE. RNA was extracted from the TH-1 cell culture supernatant using a QIAamp Viral RNA Mini Kit (Qiagen) according to the manufacturer’s recommendation. The assay was performed using a Qiagen OneStep RT-PCR Kit with 30 μl cocktails consisting of 1.2 μl Enzyme Mix, 6 μl 5X RT-PCR buffer, 6 μl 5x Q-Solution, 1.2 μl 10 mM dNTP Mix, 1.2 μl 20 μM forward (5’-GGTATAAGGCTGGTTCGGGT-3’) and reverse (5’-GCCACACCCAATTTCTCTCC-3’) primers, 8.4 μl RNase-free water, and 4.8 μl extracted RNA sample. The reverse transcription step was conducted at 50°C for 30 min, followed by an initial cycle of 15 min at 95°C, 40 cycles of denaturation at 95°C, annealing at 57°C, and extension at 72°C for 30 seconds each, and a final elongation step at 72°C for 10 min. RNase-free water was used as the negative control. PCR products were subjected to electrophoresis on a 1% agarose gel stained with ethidium bromide. The expected size band was purified using a QIAquick PCR Purification Kit (Qiagen) and sequenced in both directions on an automated ABI 3130 Genetic Analyzer (Life Technologies), to confirm that the amplified sequence was identical to the complete genome sequence determined from the MiSeq data set. RNA was extracted from samples (tissues, swabs, and bodily fluids) collected from free-ranging freshwater turtles using a QIAamp Viral RNA Mini Kit (Qiagen) according to the manufacturer’s recommendation and subjected to TFV1 screening using the RT-PCR assay. The RNA extracted from the viral-infected cell culture supernatant was used as a positive control and RNase-free water was used as negative control in every reaction. The PCR products were subjected to electrophoresis on a 1% agarose gel stained with ethidium bromide. Bands of the expected size were purified and sequenced as described above.

### Development of a RNAscope in situ hybridization assay

Select formalin-fixed paraffin embedded tissues were used in an automated *in situ* hybridization (ISH) assay to detect RNA of TFV1. Chromogenic ISH was performed on a Leica BOND RX Fully Automated Research Stainer (Leica Biosystems) using RNAscope technology [25] adapted for the Leica Systems (LS) automated staining platform [26]. A custom probe to the coding sequence of the TFV1 RNA dependent RNA polymerase (RdRP) [LS, catalog # 577138] was designed by Advanced Cell Diagnostics (Newark, California) for use on the LS platform. Blocks of formalin-fixed paraffin-embedded tissues were sectioned at 4 μm and mounted on Fisherbrand SuperFrost Plus glass slides (Fisher Scientific). Pretreatment, hybridization, signal amplification, and detection (RNAscope Reference Guide, ACD/Biotechne) were performed on the Leica BOND RX. Pretreatment conditions included sequential deparaffinization, target retrieval using Leica Epitope Retrieval Buffer 2 at 95°C for 15 min, protease digestion at 40°C for 15 min, and endogenous enzyme block. This was followed by target probe hybridization (120 min at 42°C). Signal amplification was performed through a series of reactions, i.e., RNAscope signal amplification [25], that culminated in fast red chromogenic development, and was followed by hematoxylin staining. The dihydrodipicolinate reductase (dapB) probe [LS, catalog #320758] was used as a negative control probe and was applied to separate histologic sections processed concurrently and in the same manner as those that received the TFV1 probe. As negative case controls, ISH was also performed on multiple tissues from several freshwater turtles that were not believed to be associated with epizootics, were RT-PCR negative for TFV1, and that lacked histopathological lesions provisionally associated with TFV1 infection.

## Results

### Field investigation and animal examinations

As listed in Table 2, a total of 22 turtles (89 samples), including 15 Florida softshells (*A*. *ferox*), 4 peninsula cooters (*P*. *peninsularis*), and 3 Florida red-bellied cooters (*P*. *nelsoni*), were collected from the five described study sites. Two *A*. *ferox* and one *P*. *peninsularis* were collected as carcasses, whereas the remaining 19 turtles were euthanized. Six additional samples were collected from three euthanized control turtles: *A*. *ferox* (180907–03) from Lake County, *P*. *peninsularis* (200305–02) from Lee County, and *P*. *peninsularis* (200305–03) from Brevard County.

**Table 2:** Necropsy and conventional TFV1 RT-PCR results for 25 free-ranging freshwater turtles, where SS = Florida Softshell (*Apalone ferox*), PC = Peninsula Cooter (*Pseudemys peninsularis*), and RC = Florida Red-Bellied Cooter (*Pseudemys nelsoni*). Ages are represented as adult (A) and immature (I); sexes as female (F) and male (M). Corresponding information for each study site is described in Table 1.

Animal ID^1^	Species	Collection Date	Study Site^2^	Sex	Age	Fat^3^	Weight (kg)	Plaques (O-C-P)^4^	Positive Tissues^5^	Negative Tissues^5^
180321–01	SS	3/16/18	1	F	A	4	14.9	Y-Y-X	BR, CL, KI, LI, LU, SP	HT
180321–02	SS	3/16/18	1	M	A	4	4.0	Y-Y-Y	BR, HT, KI, LI, LU, PH, PY, SP	none
180321–03	PC	3/16/18	1	F	I	2	Unk	NE-N-X	none	CL, KI, LI, SP
180329–01*	SS	3/28/18	1	M	A	3	7.2	N-N-Y	KI, SP	none
180329–02*	SS	3/28/18	1	F	A	4	5.0	N-N-X	BR, CL, HT, IN, KI, LI, LU, SP, PY	none
180419–01	SS	4/18/18	1	M	A	3	5.4	Y-Y-N	KI, SP	none
**180419–02**	SS	4/18/18	1	F	I	4	2.7	N-N-X	BR, GL, HT, IN, KI, LI, LU, SP, ST	none
180509–04	SS	5/9/18	1	M	A	2	1.6	N-N-Y	KI, LI	none
**180517–01**	SS	5/16/18	3	F	A	4	26.6	Y-Y-X	BR, KI, LI, SP	none
**180529–01**	PC	5/29/18	3	F	A	2	4.5	N-N-X	BR, SP	none
180529–02	SS	5/29/18	3	F	A	4	17.8	Y-Y-X	BR, KI	none
180907–03	SS	9/6/18	C	F	A	4	4.8	N-N-X	none	BR, SP
181102–01	SS	4/13/18	2	F	A	3	8.0	Y-Y-X	BR, CL, LU	none
**190129–01**	PC	1/28/19	4	M	A	2	3.5	Y-N-N	CS, LI, OS, SP	none
190129–02*	SS	1/28/19	4	M	A	4	6.4	Y-Y-Y	CS, OS, UR	WB
**190129–03**	SS	1/28/19	4	M	A	4	5.0	Y-Y-Y	CS, OS, SP	none
**190129–04**	SS	1/28/19	4	F	A	4	16.5	Y-Y-X	OS, SP, UR	CS, PL
190129–05*	SS	1/28/19	4	F	A	4	16.2	Y-Y-X	CS, OS, UR	WB
190226–01*	RC	2/25/19	4	M	A	2	2.2	Y-N-N	BR, LI, SP	none
190226–02*	RC	2/25/19	4	F	A	3	3.5	N-N-X	BR, SP, UR	none
190226–03*	RC	2/25/19	4	M	A	2	2.5	N-N-N	BR, KI	none
190226–04*	PC	2/25/19	4	M	A	2	3.2	N-N-N	BR, KI	none
200305–02	PC	3/3/2020	C	F	A	1	7.0	N-N-X	none	BR, KI
200305–03	PC	3/4/2020	C	F	A	1	4.4	N-N-X	none	BR, KI
200324–01*	SS	3/24/20	5	F	A	4	6.8	Y-Y-X	BR, KI, LI, SP, UR	none

^1^The nine animal IDs followed by an asterisk (*) are included in hematological analyses; aggregate results are presented in Table 3. Tissues from the 10 animal IDs in bold font were examined by *in situ* hybridization (ISH) techniques. Results are presented in Table 4.

^2^Three control animals are represented by a C.

^3^Fat Scores: 1 = none, 2 = slight, 3 = moderate, and 4 = abundant.

^4^Plaques observed on necropsy abbreviated as O-C-P (Oral-Cloacal-Phallus), where a N = no, Y = yes, NE = not examined because body part scavenged, X = female and phallus plaques not relevant.

^5^Abbreviations for tissue samples tested: BR = brain, CL = cloacal tissue, CS = cloacal swab, GL = glottis, HT = heart, IT = intestine, KI = kidney, LI = liver, LU = lung, OS = oropharyngeal swab, PH = phallus, PL = plasma, PY = pharynx, SP = spleen, ST = stomach, UR = urine, WB = whole blood.

Affected *A*. *ferox* were often found at the shoreline’s edge entirely out of the water, with their necks extended prone, or were partially submerged near shore. Moribund *Pseudemys* spp. were found similarly hauled out on banks or grasping onto items on which they would normally bask (e.g., emerging logs and floating mats of vegetation). In general, affected turtles did not attempt to flee when approached as is their normal behavior. During physical examination, turtles exhibited reduced withdrawal reflexes and were minimally responsive, permitting manipulation of the head and neck without typical defensive biting behavior. Antemortem erythema of the plastron was apparent in many *A*. *ferox*, along with sloughing of the skin, especially around the eyes. Several *A*. *ferox* had an abnormal breathing pattern whereby the turtle dorsally flexed its neck perpendicular to the ground to take a breath and then dropped its head and extended its neck, sometimes making gurgling expiratory sounds. The *Pseudemys* spp. frequently had mucoid bubbles emanating from the nares and oral commissures. Fourteen individuals (8 *A*. *ferox*, 3 *P*. *peninsularis*, and 3 *P*. *nelsoni*) had one or more ocular lesions consisting of exudative plaques on the palpebrae, opaque mucoid discharge, and corneal ulcers. In addition, the eyes of *Pseudemys* spp. were prominently sunken into the orbits.

**Fig 2:**
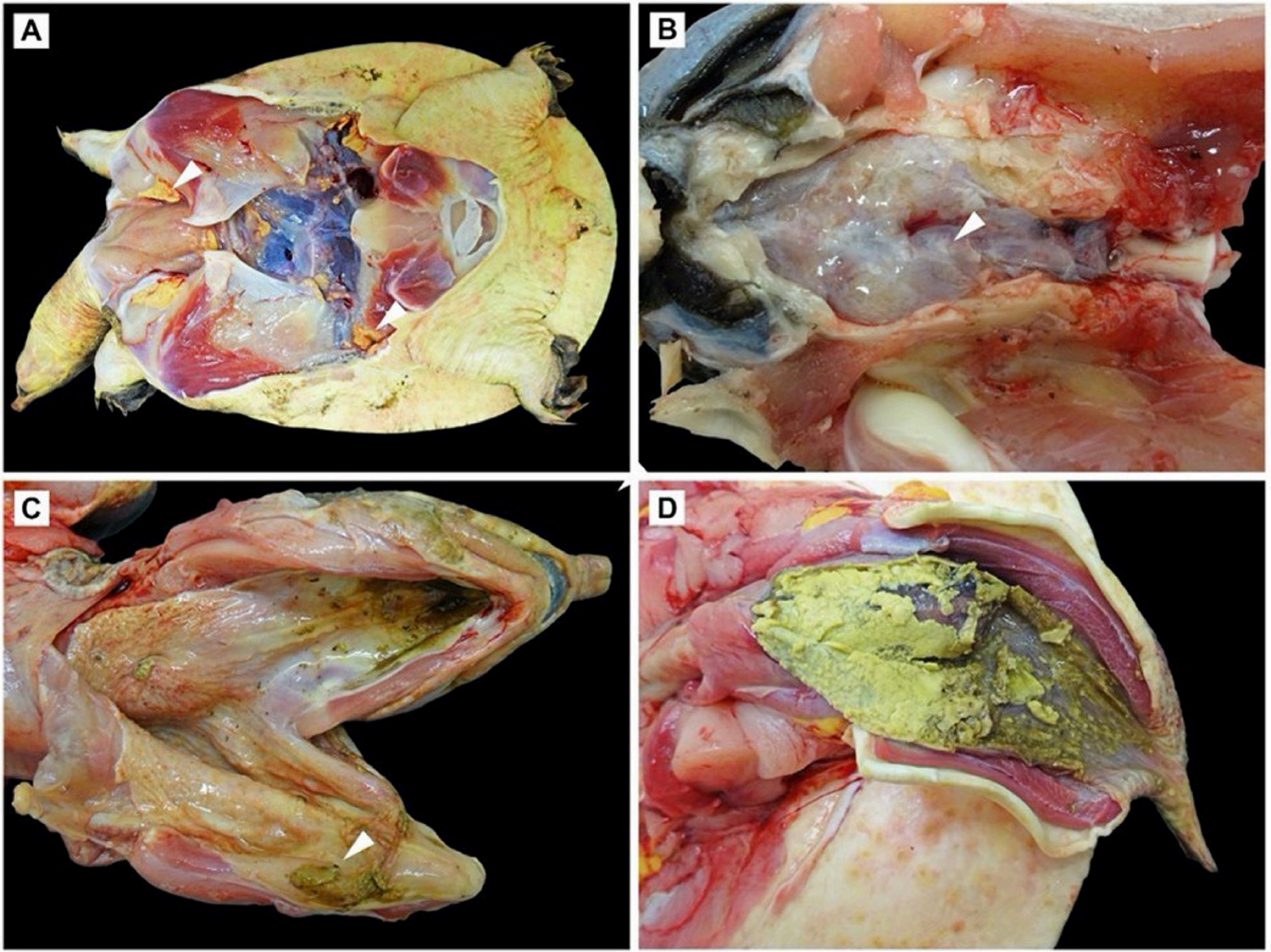
Gross observations in Florida softshell turtles (*Apalone ferox*) infected with Turtle fraservirus 1 (TFV1). A) Plastron removed showing non-atrophied fat (white arrowhead) indicating good nutritional condition. B) Milky opacity (white arrowhead) of the leptomeninges due to meningitis. C) Oral ulceration with exudative plaques (white arrowhead). D) Extensive ulceration of the phallus with exudate formation.

Consistent gross findings included exudative plaques and ulcers of the oral cavity (Fig 2) and cloaca in 11/15 (73.3%) *A*. *ferox* of both sexes (Table 2). These lesions affected the base and distal projections of the phallus in males and the urodeum around the oviductal openings of females. In contrast, cloacal plaques were not observed in cooters, and the oral plaques observed in only two individuals were relatively mild. Most softshells were in good nutritional condition, whereas body fat was atrophied in the *Pseudemys* spp., which likely contributed to the enophthalmia. Other frequent gross findings included gastrointestinal ulceration (8/22) and granuloma formation within various visceral organs.

Examination included histopathology for 17 TFV1-positive turtles, including 13 *A*. *ferox*, 2 *P*. *peninsularis*, and 2 *P*. *nelsoni*. Interpretation was often complicated by microbial infections regarded as secondary or opportunistic processes, as well as vascular lesions associated with spirorchiid trematodes (blood flukes). Twelve of 17 turtles had bacterial and/or fungal infections. Most of the bacterial infections were characterized by multisystemic granulomatous inflammation associated with intralesional bacteria or septic emboli. These infections included one softshell turtle with hepatosplenic mycobacteriosis. Fungal infections were found in two turtles with pneumonia, one turtle with severe colitis, and another turtle with mild, ulcerative dermatitis. Embolized spirorchiid ova surrounded by focal histiocytic granuloma formation was present in multiple tissues in all but 3 turtles (2 *A*. *ferox* and a *P*. *peninsularis*), often in combination with vascular lesions, which included medial hypertrophy, endarteritis, and hypertrophy of endothelial cells with formation of intracytoplasmic proteinaceous globules. The *P*. *peninsularis* without embolized spirorchiid eggs had vascular lesions suggestive of spirorchiidiasis, possibly representing a prepatent infection.

**Fig 3:**
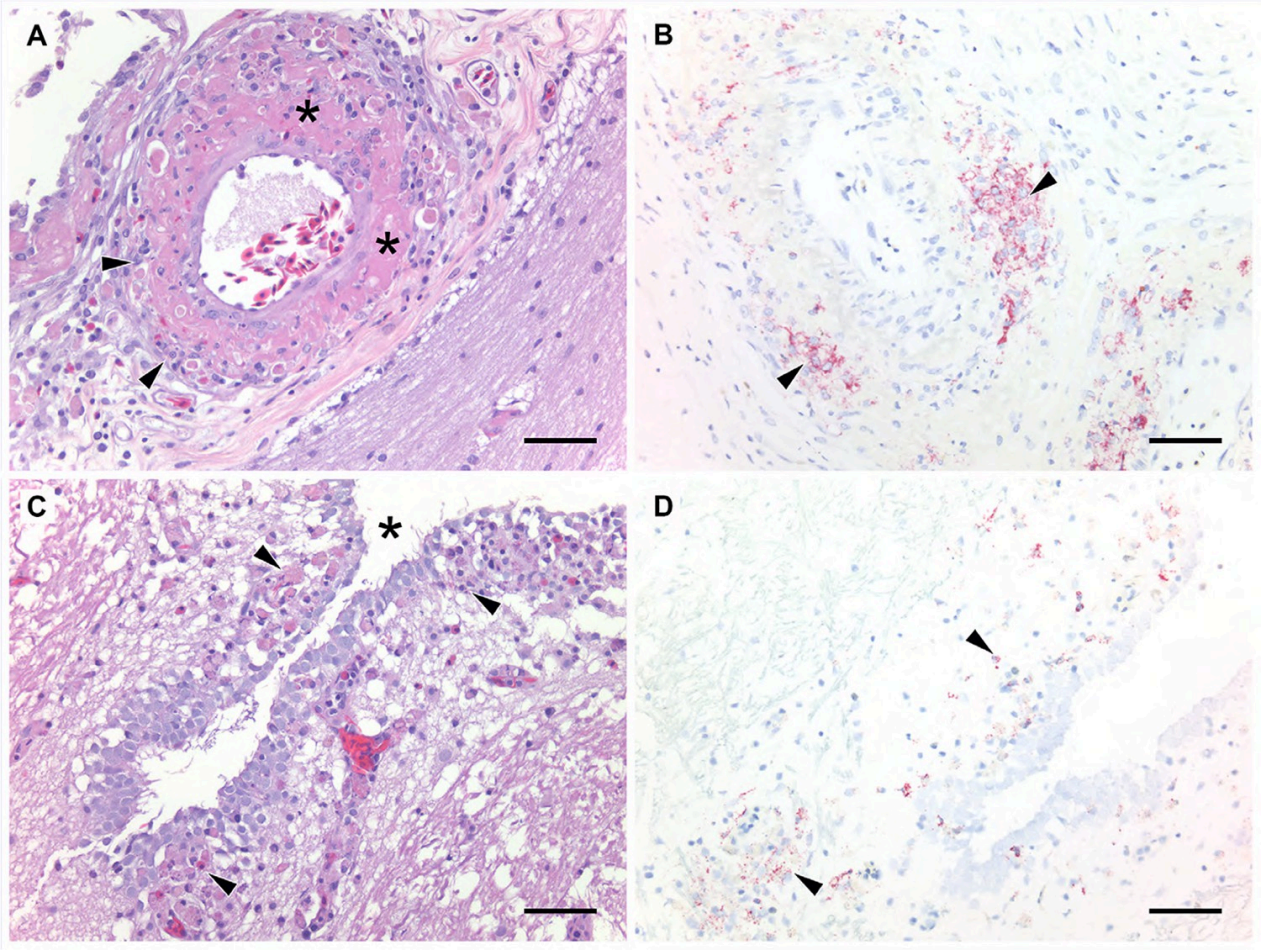
Histopathological lesions affecting the nervous system in Florida softshell turtles (*Apalone ferox*) infected with Turtle fraservirus 1 (TFV1). A) Vascular-oriented leptomeningitis with fibrinoid vascular necrosis (asterisks). Macrophages and heterophils (black arrowheads) infiltrate a vessel and the surrounding tissue. Hematoxylin and eosin. Scale bar = 65 μm. B) Leukocytes within the affected vessel wall and smooth muscle exhibit positive staining (black arrowheads) for TFV1 by RNAscope *in situ* hybridization (ISH). Scale bar = 65 μm. C) Encephalitis characterized by infiltration by heterophils and macrophages (black arrowheads) around the third ventricle (*). Hematoxylin and eosin. Scale bar = 80 μm. D) Positive staining of leukocytes and glial cells (black arrowheads) for TFV1 by ISH. Scale bar = 80 μm.

The most consistent histopathological finding in all examined TFV1 RT-PCR positive free-ranging turtles was various degrees of leptomeningitis or meningoencephalitis (Fig 3). Macrophages with fewer heterophils and lymphocytes infiltrated blood vessels and surrounding tissue, obscuring vessel walls. Vasculitis was accompanied by fibrinoid necrosis in severe cases. In those turtles with encephalitis, inflammation was vasocentric, involving the subependyma and choroid plexus. Meningitis was relatively mild in half of the examined turtles, including all of the *Pseudemys* sp.

**Fig 4:**
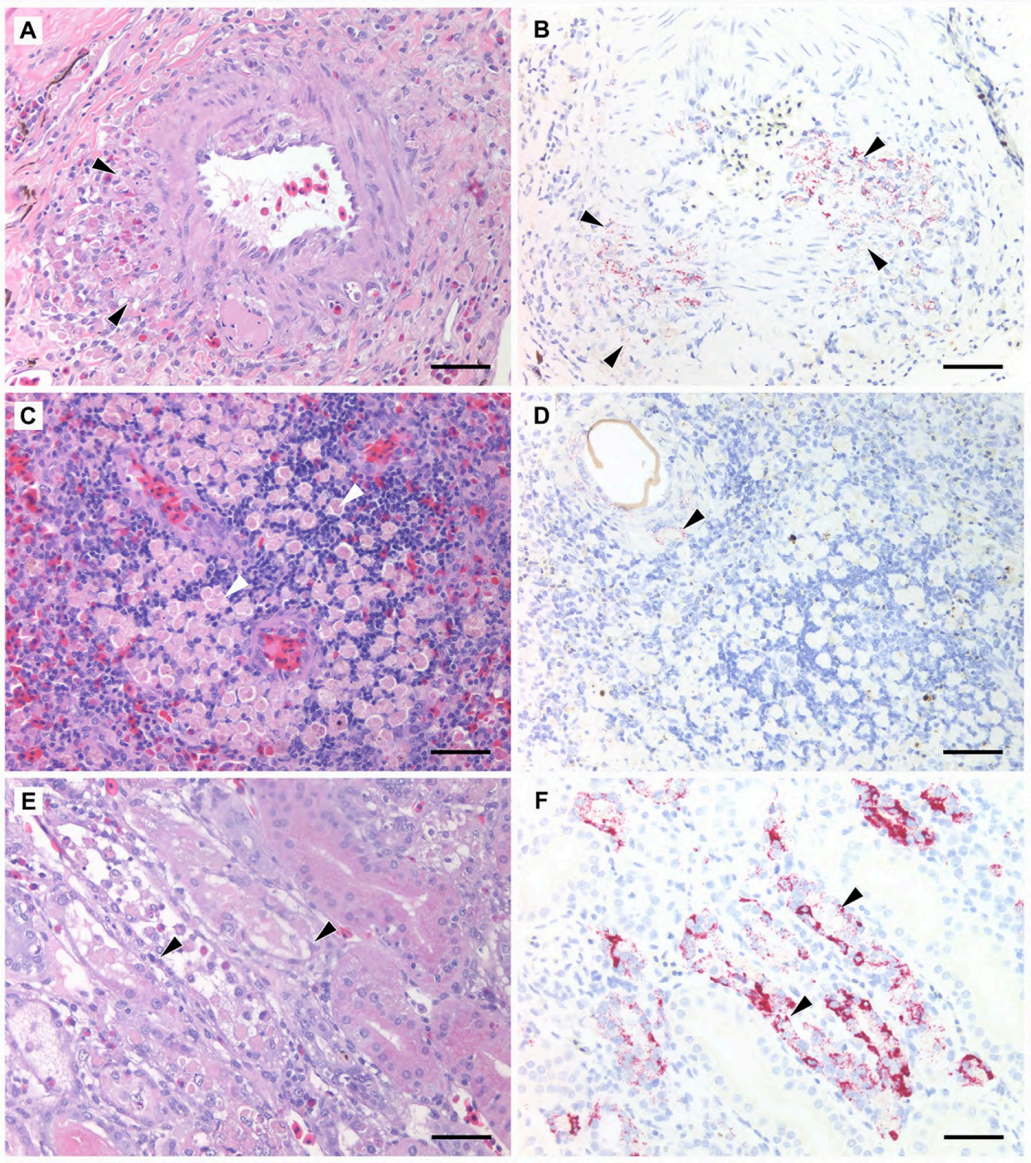
Histopathological lesions in visceral organs of Florida softshell turtles (*Apalone ferox*) infected with Turtle fraservirus 1 (TFV1). A) Vasculitis of a gastric serosal artery characterized by infiltration of the arterial wall and surrounding tissue by macrophages and heterophils (black arrowheads). The macrophages contain degenerate eosinophilic material and there are frequent necrotic cells within infiltrate. Hematoxylin and eosin. Scale bar = 75 μm. B) Many leukocytes exhibit positive labelling (black arrowheads) for TFV1 by RNAscope *in situ* hybridization (ISH). Scale bar = 75 μm. C) Collection of macrophages within the spleen in which the cytoplasm contains globular eosinophilic material and scant brown pigment granules (white arrowheads). Hematoxylin and eosin. Scale bar = 75 μm. D) There is no labelling of these macrophages for TFV1 by ISH; however, there is positive staining of macrophages (black arrowhead) and multinucleated giant cells that comprise a granuloma formed around a spirorchiid trematode egg. Scale bar = 75 μm. E) Macrophages and heterophils infiltrate renal tubules. The tubular epithelium is attenuated, and sloughed cells and leukocytes fill the lumina (black arrowheads). Macrophages contain degenerate cytoplasmic material as observed in other lesions. Hematoxylin and eosin. Scale bar = 75 μm. F) There is widespread staining of the abnormal tubular epithelium (black arrowheads) for TFV1 by ISH. Scale bar = 75 μm.

In addition to vascular-associated inflammation in the nervous system, eight turtles also had various degrees of similar vasculitis in other tissues, including the gastrointestinal tract, submucosa of oropharynx and cloaca, lung, kidney, and liver (Fig 4). These vascular changes included fibrinoid necrosis. Associated perivascular inflammation extended into the surrounding tissues, such as the pulmonary and renal interstitium. The latter was associated with renal tubular necrosis or cast formation in eight turtles.

**Fig 5:**
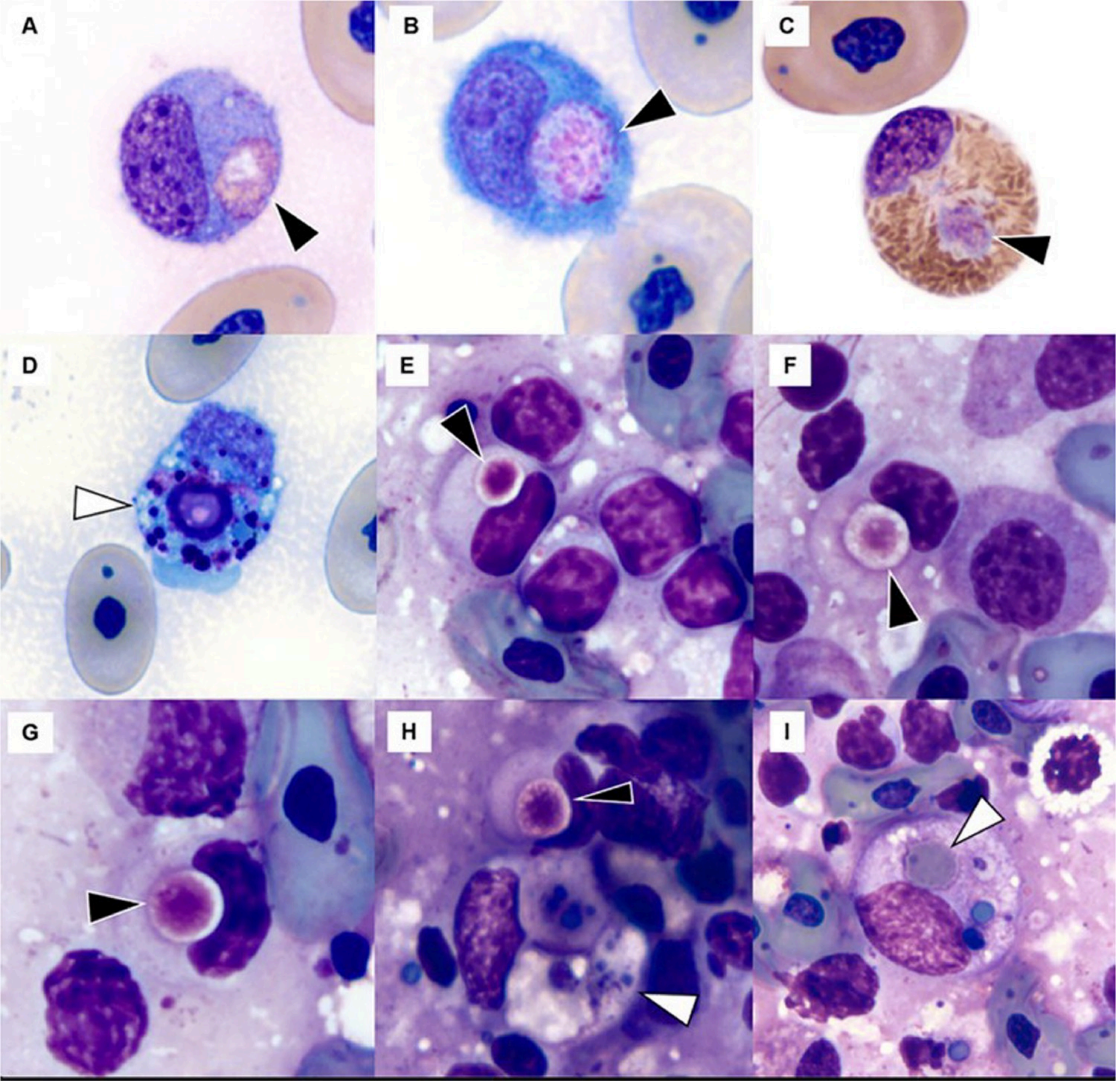
Image composite of inclusions observed in monocytes/histiocytes and heterophils of Florida softshell turtles (*Apalone ferox*) infected with Turtle fraservirus 1 (TFV1) in blood films (A-D) and splenic tissue imprints (E-I). Black arrowheads show inclusions of potential viral origin or viral-associated protein; white arrowheads indicate phagocytic activity. A-B) monocytes, C) heterophil, D) monocyte with non-specific cellular or nucleoproteinaceous debris, H-I) histiocytes with white arrowheads showing intracytoplasmic greenish-blue pigment suggestive of hemosiderin with I) also including a fragment of an erythrocyte in addition to presumptive hemosiderin. Wright-Giemsa stain, 100x objective.

Another consistent observation among TFV1 positive turtles was alteration of macrophage morphology (Fig 5). The cytoplasm of macrophages within most lesions contained abundant non-pigmented and some pigmented degenerate material. Collections of these cells within the spleen, associated with overt splenitis in some cases, was a prominent feature in all positive turtles. Cytological examination of affected macrophages in tissue imprints of liver and spleen from a softshell turtle showed increased histiocytic activity with distinct pale eosinophilic, round granular inclusions with morphology suggesting potential viral origin or viral-associated protein (Fig 5). Other relevant cytological observations in histiocytes included phagocytosis of erythrocytes, greenish-basophilic material most consistent with hemosiderin, melanin, or cellular/nucleoproteinaceous debris.

The ulcerative lesions involving the oropharynx, cloaca, phallus, and eyelids were characterized by intense heterophilic inflammation containing numerous surface bacteria. Vasculocentric, predominantly histiocytic inflammation, as previously described, was observed within the underlying submucosa and was a prominent feature in ulcerative lesions involving the phallus. No inclusion bodies or syncytia were observed in the affected epithelia.

Hematological data of nine turtles are presented in Table 3. Leukogram findings included chronic-active inflammation in all turtles with available blood film review, characterized by leukocytosis (N = 8), heterophil left-shift (N = 9) with mild to moderate toxic change (N = 6), monocytosis (N = 6), and presence of plasma cells (N = 3) or melanomacrophages (N = 2). Heteropenia with left-shift consistent with overwhelming inflammation was observed in one turtle. Pale eosinophilic, round to oval granular inclusions suggestive of potential viral origin or viral-associated protein similar to those found in tissue imprints were observed in monocytes and rarely in heterophils in three of nine turtles (Fig 5). Monocytes were frequently reactive in all turtles and occasionally exhibited non-specific phagocytosis of cellular or nucleoproteinaceous debris. Thrombocytes were adequate.

**Table 3:** Hematological data of freshwater turtles (N = 5 *Apalone ferox*; N = 4 *Pseudemys* spp.) infected with Turtle fraservirus 1. RBC = red blood cells. NDA = no data available.

Analyte	Unit	n	Mean (Minimum-Maximum)	Mean^[^^27^^]^ (Minimum-Maximum) Western pond turtles (N = 20)
Packed cell volume	%	2	24(23–25)	25(19–32.5
Immature RBC	#/100 mature RBC	9	2(0–7)	NDA
White blood cell estimate	K/μl	9	40.44(17.80–65.00)	12.96(6.80–24.00)
Mature heterophils	K/μl	9	10.06(0.00–21.00)	2.56(0.66–5.32)
Immature heterophils	K/μl	9	8.20(2.60–21.00)	NDA
Lymphocytes	K/μl	9	6.20(0.93–10.00)	3.34(0.11–12.18)
Monocytes	K/μl	9	11.38(2.20–28.00)	0.67(0.21–2.06)*
Eosinophils	K/μl	9	0.76(0.00–3.30)	4.10(1.90–7.92)
Basophils	K/μl	9	3.70(1.40–7.70)	2.30(0.64–6.48)
Hemogregarines	#/100 RBC	9	1(0–11)	NDA
Total protein by refractometer	g/dl	2	5.6(5.4–5.8)	NDA

*Monocytes and azurophils combined.

Plasma chemistry, blood culture, and lead concentration were performed in two representative affected softshell turtles that were subsequently euthanized. Blood chemistry abnormalities are summarized with pertinent necropsy diagnoses. Both turtles had hyperuricemia suggestive of renal disease and/or dehydration (uric acid 12.9 and 17.7 mg/dl), which was attributable to tubulointerstitial nephritis with cast formation. Both also had hypocholesterolemia (both < 50 mg/dl) and one had hypotriglyceridemia (29 mg/dl) consistent with reduced feeding (empty digestive tracts). Another consistent, but nonspecific finding was markedly increased creatine kinase (21,434 U/L and 33,555 U/L) and mildly increased aspartate aminotransferase (AST) activities (281 U/L and 710 U/L). These elevations are attributable to exertion from capture and/or handling; one turtle also had myositis and muscle necrosis that likely was contributory. Blood culture from one softshell turtle grew various organisms, including *Aeromonas hydrophila* subsp. *hydrophila*, *Morganella morganii* subsp. *morganii*, and a 3^rd^ Gram-negative bacillus. No bacterial growth was detected on blood culture from the second turtle. Lead concentration was non-detectable in both turtles.

No additional information was available from the farmed softshell turtle epizootic in 2007. Thus, the Florida facility could not be contacted to determine the facility layout and husbandry practices, the time of year and extent of the disease episode, number of species on the farm, or how many were affected. Moreover, we could not ascertain whether the facility sourced turtles from the wild as a ranching operation, propagated turtles from wild stock, and/or acquired turtles through commercial sources. Notably, two separate mortality events involving wild softshell turtles in Florida were reported by the public to FWC in May and August 2007. Animal examinations were not possible in either case due to advanced decomposition.

### Virus isolation

**Fig 6:**
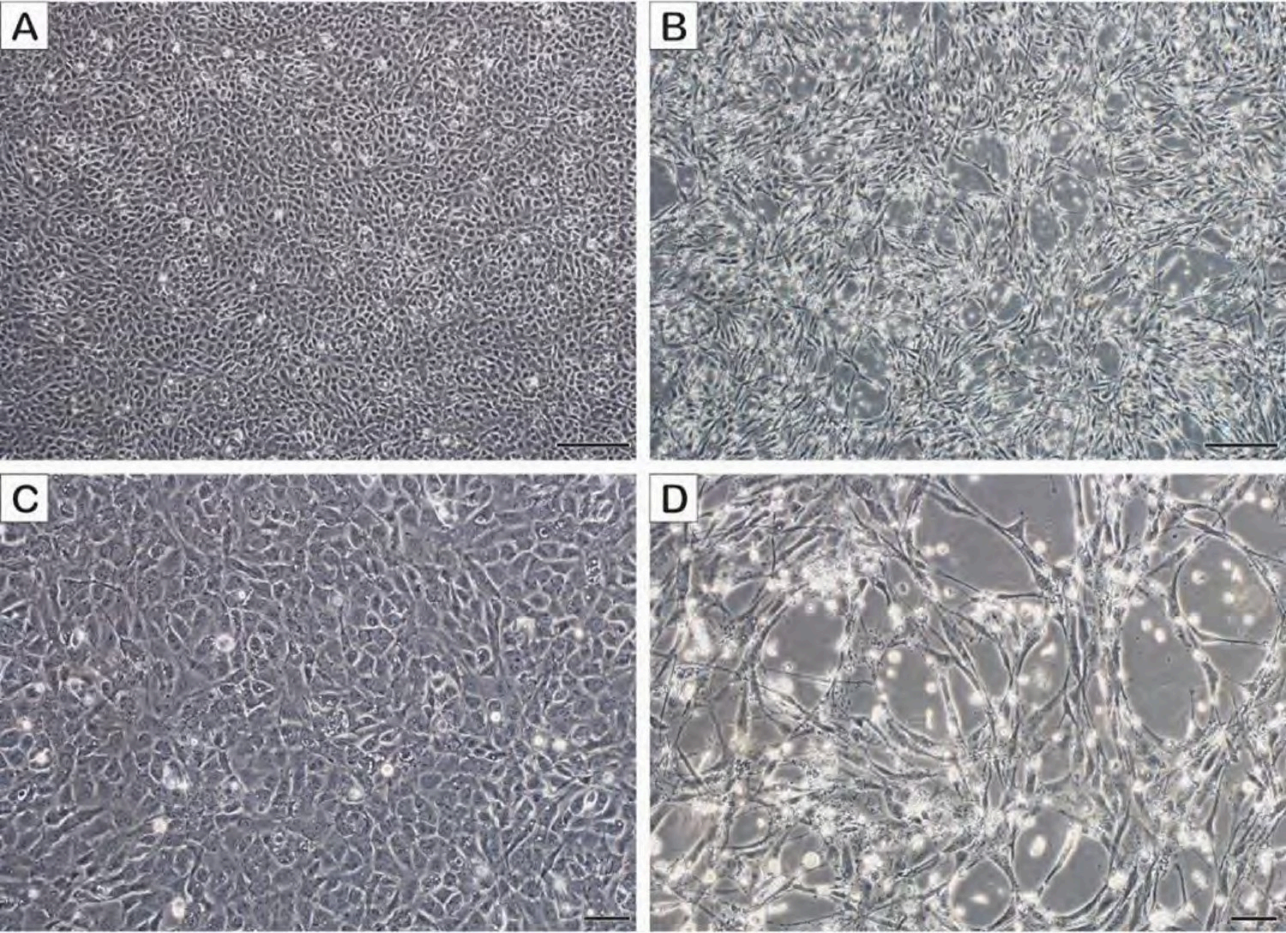
*In vitro* growth characteristics of the Turtle fraservirus 1 in Terrapene heart (TH-1) cells (viral passage 2). A) and C) uninfected TH-1 cells, 8 days post-inoculation (dpi). B) and D) appearance of plaques within the TH-1 monolayer 8 dpi. Affected cells appear refractile and either elongated and spindly or rounded. Scale bars in A and B = 200 μm, C and D = 50 μm.

Cells within each of the 2020 TH-1 cultures displayed increased vacuolation 7–12 dpi, as compared to controls. Vacuolation was observed earliest and became most pronounced in the brain and urine cultures at 12 dpi. All cultures were passaged and plaques were observed in all VP2 cultures by 6 dpi. Plaques began as foci of spindly (thin and elongated) TH-1 cells that then rounded and became refractile. The CPE was most dramatic in the VP2 urine cultures in which plaques were observed to coalesce until the entire monolayer was affected by 8 dpi (Fig 6). CPE from the softshell turtle internal tissue homogenate in 2007 was similar, except that the formation of coalescing plaques was observed in VP1.

### Transmission electron microscopy (negative staining)

**Fig 7:**
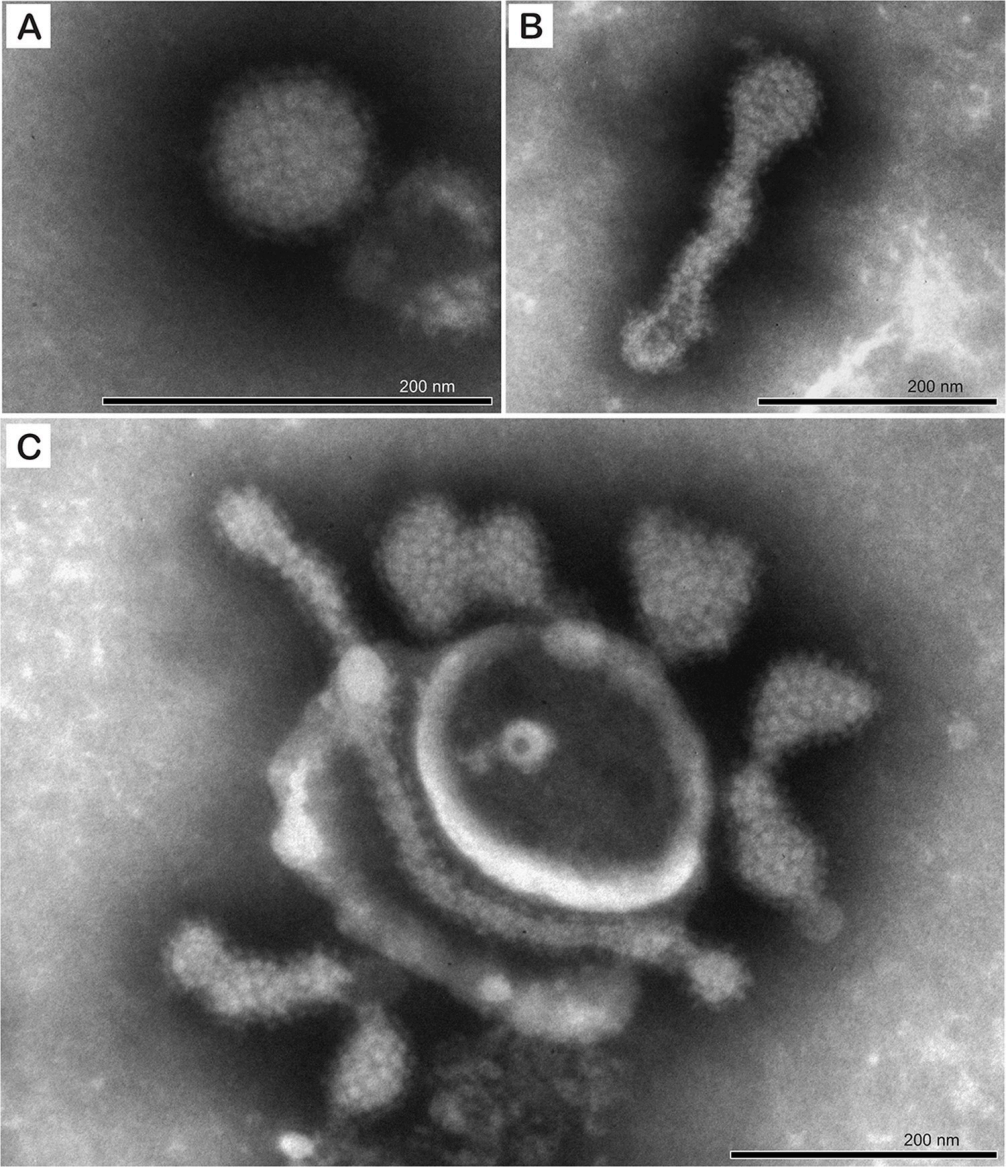
Negative stain electron photomicrograph illustrating the ultrastructural features of the Turtle fraservirus 1 (TFV1) virion. A) spherical particle with prominent glycoprotein spikes. B and C) pleomorphic particles including elongated forms. Scale bars = 200 nm.

In negatively stained preparations of cell culture supernatants from 2007 infected TH-1 cells, highly pleomorphic particles (round, oval, elongated, irregular shaped) were observed (Fig 7). The pleomorphic and filamentous particles were measured 99–194 nm in diameter and 221–553 nm long, respectively. All particles displayed regular surface subunit structures 11.0 nm (N = 10, SD = 1.13 nm) in diameter and a prominent globular fringe indicative of an enveloped virus.

### Transmission electron microscopy (ultrathin sections)

**Fig 8:**
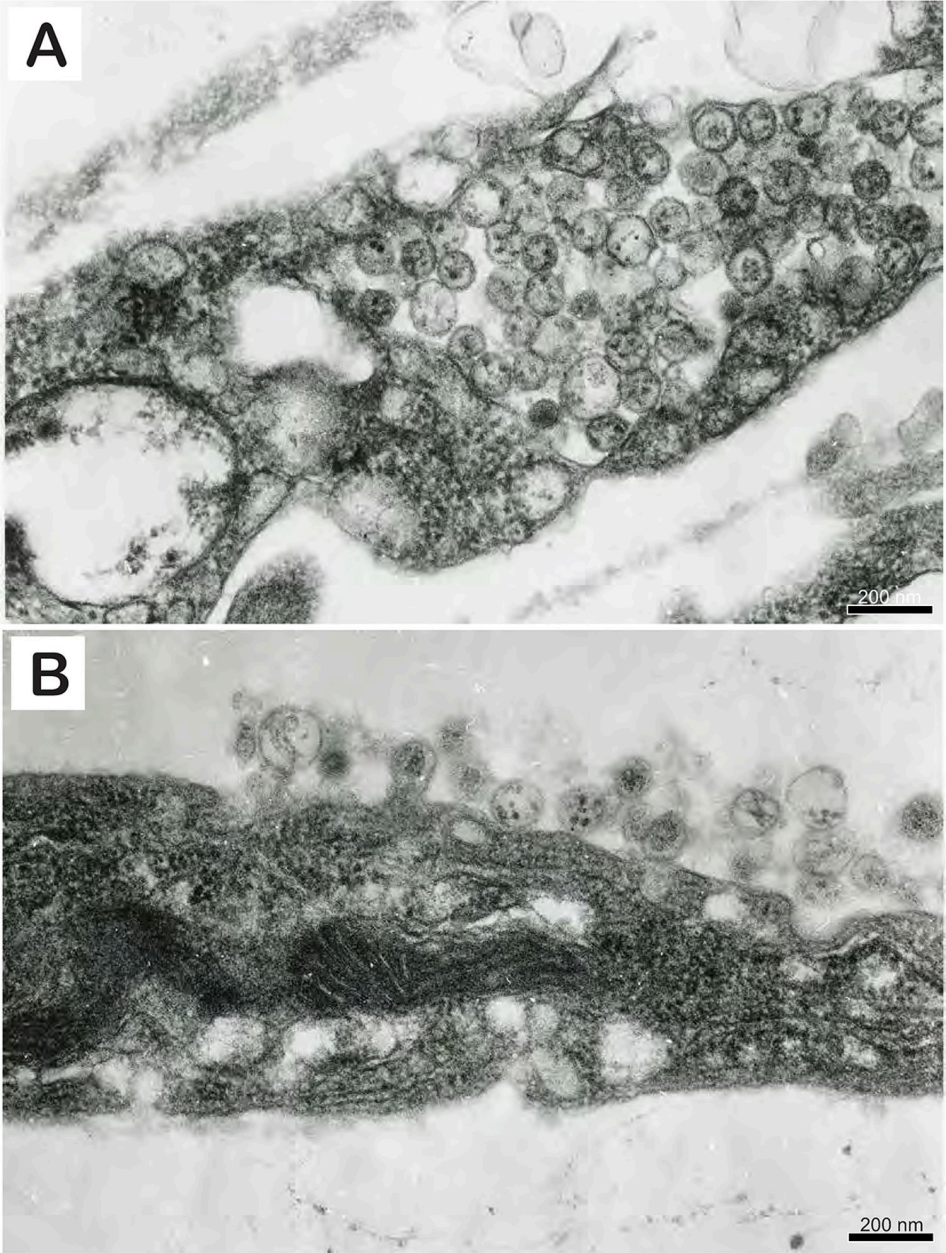
Thin section electron photomicrograph illustrating: A) a cluster of round to oval virus particles present in a cytoplasmic vesicle of an infected Terrapene heart (TH-1) cell. B) Turtle fraservirus 1 particles budding from the surface of an infected TH-1 cell and the presence of extracellular pleomorphic virions.

In ultrathin sections of infected TH-1 cells mostly round or slightly oval virus particles 110–125 nm in diameter were observed in either cytoplasmic vacuoles (Fig 8A) or at the cell surface with some of them budding from it (Fig 8B). Several intracellular and extracellular virions contained characteristic electron-dense granules 12–21 nm in diameter compatible in size with host cell ribosomes.

### Comparative genomic and phylogenetic analyses of TFV1

**Fig 9:**
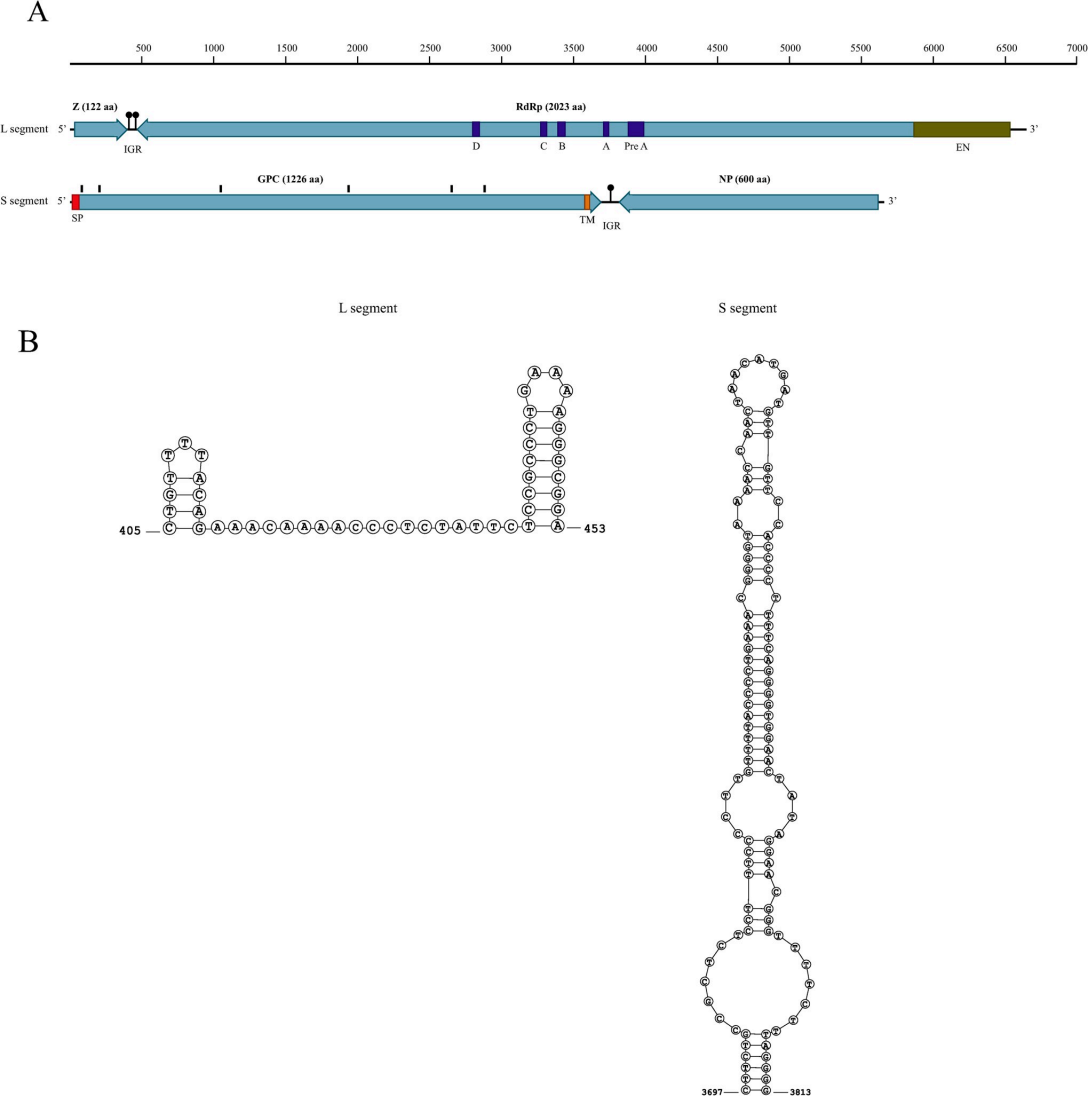
Genomic characterization of the 2020 isolate of Turtle fraservirus 1 (TFV1). (A) Schematic representation of the TFV1 genome. Open reading frames (ORFs) are shown as light blue boxes, the endonuclease domain in the RNA-dependent RNA polymerase is indicated by an olive green box and the conserved motifs are indicated by dark blue boxes. The glycosylation sites in the glycoprotein precursor polyprotein are marked by black lines above the ORF, the signal peptide is indicated by a red box and the transmembrane domain is indicated by an orange box. Abbreviations: Z, zinc-finger protein; RdRP, RNA-dependent RNA polymerase; GPC, glycoprotein precursor polyprotein; N, nucleoprotein; IGR, non-coding intergenic region; EN, endonuclease; Pre A, premotif A; A, motif A; B, motif B; C, motif C; D, motif D; SP, signal peptide; TM, transmembrane domain. (B) Predicted stem-loop structures within the non-coding intergenic regions in L and S segments.

The genomes of the 2007 and 2020 TFV1 isolates were determined to be bi-segmented with an ambisense coding arrangement without polyadenylated tracts at the 3′-termini. The 5′- and 3′-terminal tetranucleotides of the L segment were complementary to each other in the 2020 isolate. In the 2007 isolate, the 5′- and 3′-terminal tetranucleotides were complementary to each other and identical to the 2020 isolate; however, the last two tetranucleotides at the 3’terminus could not be determined from the sequence data. For both isolates, the 5’-end sequences on the S segment were not determined; whereas, the tetranucleotides at the 3’-end were identical to the 3’-terminal sequence of the L segment of the 2020 isolate. The L segments of 2007 and 2020 isolates were 6,645 and 6,647 nucleotides in length, respectively. The isolates exhibited 99.26% nucleotide sequence identity to each other and encoded two proteins of 2,023 amino acids and 122 amino acids in the sense and antisense strands, respectively (Fig 9A). None of these proteins showed detectable similarity to any protein sequence when compared to the sequences of RNA virus proteins using BLASTP and to the database of protein domain profiles using HHPred. However, given the apparent similarity of the TFV1 genome organization to that of arenaviruses, we suspected that the larger protein encoded in the sense orientation in the L segment was the virus RNA-directed RNA polymerase (RdRP). To test this prediction, we compared the sequence of this protein to the collection of previously developed profiles for different families of viral RdRPs [20]. Significant similarity was detected to several profiles, but a full length alignment was obtained only with the generic profile for *Negarnaviricota* RdRPs, in agreement with the prediction that this largest TFV1 protein contained a highly diverged RdRP. The counterparts to the conserved RdRP premotif A, and motifs A, B, C, and D were identified in the predicted TFV1 RdRP by the examination of the multiple alignment with other negarnavirus RdRPs (S1 Fig). The small protein that is encoded in the antisense strand of the L segment did not show significant similarity to any proteins. However, examination of the protein sequence identified a Cx3C… Hx3H (x denotes any amino acid) motif that might represent a metal-binding site (S2 Fig). Therefore, it appears likely that, by analogy with the genome organization of mammarenaviruses and reptarenaviruses, this is the Zn-binding matrix protein (Z) of TFV1.

In the 2020 isolate, the S segment of the TFV1 genome was 5,658 nucleotides in length and encoded two predicted proteins of 600 and 1226 aa in the sense and antisense strands, respectively (Fig 9A). The S segment of the 2007 isolate was 5,642 nucleotides in length, encoded two predicted proteins of 600 and 1225 aa (missing the predicted start codon [methionine] present in the 2020 isolate) in the sense and antisense strands, respectively, and exhibited 98.56% nucleotide sequence identity to the 2020 isolate. The C-terminal half of the larger protein showed highly significant sequence similarity (HHPred probabilities 96–97) to the envelope glycoproteins of a variety of negative sense RNA viruses with segmented genomes, including tenuiviruses and hantaviruses. Thus, this protein is the glycoprotein precursor (GPC) of TFV1. A signal peptide, a transmembrane domain, and eight glycosylation sites were predicted in GPC (Fig 9A). The 600 aa protein encoded in the sense strand of the S segment was not significantly similar to any protein sequences. However, again, by analogy with the genome organization of arenaviruses, it seems likely that this protein is the TFV1 nucleoprotein (N). In both L and S segments, the open reading frames were separated by non-coding intergenic regions containing one or more predicted stable stem-loop (hairpin) structures that are likely to function as transcription terminators (Fig 9B). Excluding the L and S segments contigs, 25 contigs with >90% nucleotide sequence identity were present in both the 2007 and 2020 TFV1 isolate assemblies. All 25 contigs displayed the highest nucleotide sequence identity to eukaryotic sequences (e.g., turtle) that were likely derived from the Terrapene heart cell line. Additional TFV1 segments were not detected.

**Fig 10:**
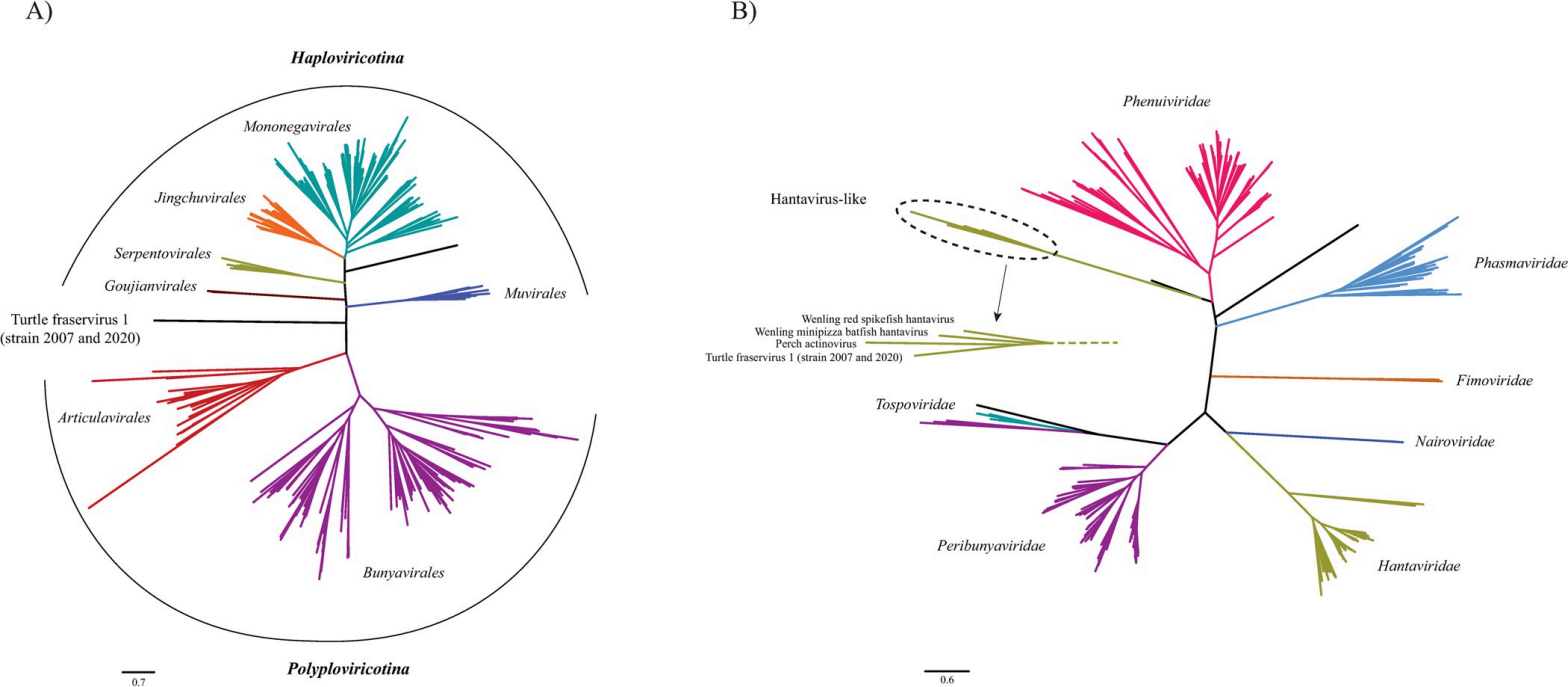
Phylogenies of the RdRP and GPC of the Turtle Fraservirus 1. A) Maximum likelihood tree (inferred with IQ-tree and the rtREV+F+R9 model) constructed from a collection of custom profiles for the RdRPs of all known negarnaviruses. Only the 7 order-level clades are shown (families and species within each clade are collapsed). B) Maximum likelihood tree (inferred with IQ-tree and the WAG+F+R9 model) constructed from a collection of bunyavirus-like glycoproteins. Only the 7 family-level clades are shown (species within each clade are collapsed). Scale bars indicate average amino acid substitutions per site. Both trees are unrooted.

We constructed maximum likelihood phylogenetic trees for the two proteins of TFV1, for which homologs were identified in other viruses of the phylum *Negarnaviricota*, namely RdRP and GPC. In the RdRP tree, TFV1 joined the clade separating the subphyla *Haploviricotina* and *Polyploviricotina* (Fig 10A), that is, the deepest division in *Negarnaviricota*. Thus, apart from the inclusion in the phylum *Negarnaviricota*, TFV1 RdRP failed to show phylogenetic affiliation with any known group of viruses. The GPC phylogeny presented a sharp contrast to that of the RdRP. In the GPC tree, TFV1 robustly clustered with the homologs from a distinct group in the family *Hantaviridae*, fish hantaviruses of the genus *Actinovirus* that have been recently discovered by metagenomic analysis (Fig 10B).

### Development of a reverse transcription PCR assay

A total of 80 samples collected from 21 free-ranging freshwater turtles were positive by the TFV1 RT-PCR assay developed in this study (Table 2). For all positive samples, the partial RdRP sequences were identical to the two TFV1 sequences assembled from the Illumina MiSeq data. Each TFV1-positive turtle had between two to nine positive samples by RT-PCR. Among the tissues tested, samples of spleen (N = 14), brain (N = 13), liver (N = 9), kidney (N = 12), lung (N = 5), cloaca (N = 3), heart (N = 3), pharynx (N = 2), intestine (N = 2), stomach (N = 1), glottis (N = 1), and phallus (N = 1) tested positive. The TFV1 RNA was also successfully amplified by RT-PCR from oral (N = 5) and cloacal (N = 4) swab samples, as well as from urine (N = 5). The only turtle associated with the epizootic that was negative by RT-PCR was an immature peninsula cooter (180321–03) that had been predated and lacked histopathologic lesions suggestive of viral infection.

### Development of a RNAscope in situ hybridization assay

TFV1 RNA was detected in multiple cell types for all three species and was consistently associated with neurological lesions and vasculitis in all cases. No staining was observed in multiple tissues from any of the three negative control turtles. The positive staining was detected in macrophages, ependymal cells, glial cells, endothelium, and vascular smooth muscle (Figs 3 and 4, Table 4). The macrophages with degenerate morphology were often positive within vascular lesions. Notably, virus was not detected in the distinct collections of macrophages in the spleen with the exception of a *P*. *nelsoni*, which had a diffuse, intense staining of macrophages circulating within the red pulp. Macrophages comprising granulomas in response to endoparasites, especially those formed around spirorchiid eggs, also exhibited positive staining, particularly near the center of the granulomas (Fig 4). Similar positive staining was observed in a pulmonary fungal granuloma in an *A*. *ferox*. Some blood vessels and histiocytic infiltrates within the submucosa of the pharynx and cavernosum of the phallus underlying or adjacent to ulcers also exhibited staining. We did not detect virus RNA in the genital or cloacal mucosa. Intense staining of the renal tubular epithelium was observed in three *A*. *ferox* and one *P*. *peninsularis* and was associated with tubular necrosis and/or tubulointerstitial nephritis, and formation of cellular casts (Fig 4). Oviduct was opportunistically included in one turtle and had intense staining of the mucosa. Lastly, we observed intense staining of hepatocytes without any evident hepatocellular lesions in one *P*. *nelsoni*. Cell types in which we did not detect virus included the respiratory and gastrointestinal mucosal epithelium, myocardium, gonads, and adrenal glands.

**Table 4:** *In situ* hybridization (ISH) results for tissues^1^ examined from 10 TFV1 RT-PCR positive, and three TFV1 RT-PCR negative, freshwater turtles. Results are expressed as negative, ISH [–] or positive with the staining intensity subjectively indicated by the number of plus signs. none = no tissues qualified under the column heading category.

Animal ID	Species^2^	ISH [–]	ISH [+]	ISH [++]	ISH [+++]
180329–01	SS	GB, TB, UB	PA, HT, IT, LI, OM, SP, TE	BR, KI, LU, PH, PY, ST	none
180329–02	SS	none	HT, SP	BR	none
180419–02	SS	none	BR, HT, LU	KI	none
180517–01	SS	none	HT	none	BR, KI
180529–01	PC	none	HT	BR, KI	none
180907–03	SS	BR, HR, SP	none	none	none
190129–01	PC	HT	AP, BR	none	none
190129–03	SS	AD, PY	BR, OM, PH, SP, ST	LI	KI, LU
190129–04	SS	AD, CL, OV, PA	IT, KI, LI, LU, PY, SP	BR	none
190226–01	RC	none	none	SP	BR, LI
190226–02	RC	none	LI, SP	BR	none
200305–02	PC	BR, CL, HT, IT, KI, LI, LU, OV, PA, PY, SP, TB	none	none	none
200305–03	PC	BR, HT, IT, KI, LI, LU, OV, PY, SP, ST, TB	none	none	none

^1^AD = adrenal, AP = adipose, BR = brain, CL = cloaca, GB = gall bladder, HT = heart, IT = intestine, KI = kidney, LI = liver, LU = lung, OM = oral mucosa, OV = oviduct, PA = pancreas, PH = phallus, PY = pharynx, SP = spleen, ST = stomach, TB = trachea/bronchus, TE = teste, UB = urinary bladder

^2^SS = Florida softshell (*Apalone ferox*), PC = Peninsula cooter (*Pseudemys peninsularis*), and RC = Florida red-bellied cooter (*P*. *nelsoni*).

## Discussion

In this study, we characterized a virus isolated from diseased captive freshwater turtles from a Florida turtle farm in 2007 and from an ongoing epizootic affecting free-ranging populations of multiple native Florida freshwater turtle species that was first recognized in the Spring of 2018. Challenge studies are needed to determine the route(s) of transmission of TFV1, its host range, and its pathogenicity to different turtle species in fulfillment of River’s postulates. However, we contend that the presented data support TFV1 as a primary pathogen and the likely cause of lethal systemic disease capable of causing epizootics among freshwater turtles. Evidence of disease causation include common clinical and pathological findings among affected turtles in junction with consistent detection of viral RNA in tissues, fluids, and exudates using a conventional RT-PCR assay, labeling of virus within lesions by *in situ* hybridization, and absence of viral detection in conspecific controls without lesions using these methods.

The growth of the turtle virus in TH-1 cells facilitated its downstream ultrastructural and genomic characterization. TFV1 shares several features in common with members of the family *Arenaviridae* including a genome with: 1) a bi-segmented structure with ambisense coding arrangement without polyadenylated tracts at the 3′-termini in both L and S segments, 2) the L segment encoding the RdRP and a putative zinc-binding matrix protein present in mammarenaviruses and reptarenaviruses, 3) the open reading frames of both the L and S segments are separated by non-coding intergenic regions consisting of one or more stem-loop (hairpin) structures. Also, like arenaviruses, the turtle virus virion is round to pleomorphic, possesses an envelope with prominent spikes acquired by budding through the host cell membrane, and contains darkly staining granules (Latin: arena, sand) that presumably represent ribosomes acquired from the host cell.

However, sequence comparisons and phylogenetic analysis did not identify TFV1 as a member of the family *Arenaviridae* or any other known virus family, but rather presented a more complex picture of its evolutionary relationships. The RdRP of TFV1 is extremely divergent so that even its identification as belonging to a negarnavirus presented a challenge. Nevertheless, using our custom collection of profiles for all known groups of RdRPs, this assignment was made confidently and is also supported by the genome organization. In accord with the observed extreme sequence divergence in the phylogenetic tree of the RdRPs, TFV1 showed no affinity with any particular group within the phylum *Negarnaviricota*, but rather formed a distinct clade at the same depth as the two subphyla, *Haploviricotina* (negarnaviruses with monopartite genomes) and *Polyploviricotina* (negarnaviruses with multipartite genomes). Given that the RdRP, the only universally conserved protein in RNA viruses, is the basis of the taxonomy above the family level, TFV1 clearly could not be included in any of the established negarnavirus families. We propose to create the family *Tosoviridae* in recognition that this virus in free-ranging turtles was first detected in the Tosohatchee Wildlife Management Area. We suggest the name Turtle fraservirus 1 (TFV1) to serve as the type species within the genus Fraservirus (in honor of Woody Fraser who first isolated and characterized the ultrastructural features of this virus in 2007). Given the deep placement of TFV1 RdRP in the phylogeny, it appears likely that once more tosoviruses are discovered, this group will attain a higher taxonomic rank, at the order or a class level.

The only other TFV1 protein with detectable homologs, GPC, shared a far greater sequence similarity with GPCs of a variety of negarnaviruses. Furthermore, in the phylogenetic tree of the GPCs, TFV1 confidently grouped with fish hanta-like viruses of the genus *Actinovirus* within the family *Hantaviridae*. In principle, the most facile route of evolution for viruses with segmented genomes, in which different genes have distinct provenances, appears to be segment reassortment. However, the genome organization of TFV1 (and arenaviruses) differs from that of hantaviruses including those in the genus *Actinovirus* [28]. Like arenaviruses, TFV1 encompasses two genome segments, such that the GPC and the (putative) NP are encoded in different orientations on the same segment. In contrast, hantaviruses possess three genome segments so that GPC and NP are encoded in M and S segments, respectively. Thus, TFV1 could have evolved by segment reassortment between an uncharacterized virus, with the L segment coming from a distinct, so far undetected group of negarnaviruses, and the S segment derived from another unknown virus encoding a GPC related to those of aquatic hanta-like virus. Alternatively, the ancestor of TFV1 might have acquired the GPC gene from an actinovirus via intrasegment recombination. Evolution of new, large groups of viruses by segment reassortment has been reported for positive sense RNA viruses, in the case of ourmiaviruses that combine an RdRP originating from capsid-less narnavirus with a capsid protein originating from a tombus-like virus [29]. To our knowledge, however, such chimeric evolutionary scenario has not been reported for any of the major groups of negarnaviruses. Intrasegment recombination is the likely evolutionary scenario for snake arenaviruses that might have acquired the GPC from a filovirus [30]. A better understanding of the contributions of these different ways of mixing and matching of negarnavirus genes should come from a broader study of viruses infecting diverse hosts.

Experimental infections are necessary to better understand TFV1 tissue tropisms, disease expression, and transmission. Many affected turtles had secondary microbial infections and vascular injury from spirorchiidiasis, which complicated attribution of lesions to specific etiology. Moreover, some lesions such as oral, conjunctival, or genital ulceration are inherently nonspecific. The most diagnostically informative histopathological findings associated with TFV1 was vascular-oriented meningitis or meningoencephalitis, systemic vasculitis, and altered macrophage morphology, with the recognition that these lesions may be very subtle in emydidae species. In blood films and cytological specimens, intracytoplasmic inclusion bodies within macrophages were also useful for detecting TFV1 infection. We hypothesize that TFV1 either infects mucosae through horizontal transmission or is inoculated by a vector. Viral tropism for macrophages is likely key to systemic spreading of the virus and disease expression. Mucosal lesions may result from either mucosal or vascular injury or opportunistic infections predisposed by viral immunosuppression, which facilitates other secondary infections as well. In addition, detection of TFV1 within the renal tubular epithelium, as well as TFV1 RT-PCR positive urine samples, suggests that urinary excretion also may play a role in transmission.

Most members of the order *Bunyavirales* are transmitted to terrestrial animals and plants via blood-sucking or sap-sucking arthropods and rodents. However, the transmission of aquatic animal bunyaviruses of crustaceans, fish, and aquatic chelonians remains poorly understood. One of the better studied examples of a crustacean-infecting bunyavirus is the crab haemocytic virus (CHV) that infects European shore crabs (*Carcinus maenas*) [31,32]. Infected crabs exhibit abnormal clotting of the hemolymph and haemocytopoenia and the disease can be transmitted to uninfected crabs by injecting them with the filtered hemolymph of CHV-infected crabs [33]. Another crustacean bunyavirus infecting cultured redclaw crayfish (*Cherax quadricarinatus*) in Queensland, Australia can be transmitted from diseased to healthy individuals by cohabitation [34]. Although a peribunyavirus was recently sequenced following its isolation from free-ranging largemouth bass, the authors did not determine how it was transmitted or its pathogenic potential [35]. Interestingly, a virus argued to be a bunyavirus, based on its serological cross-reactivity to peribunyaviruses, was isolated and passaged in suckling mice following intracerebral injection of a blood clot homogenate from an apparently healthy free-ranging Texas spiny softshell turtle (*A*. *spinifera emoyri*) in Texas in the early 1970s [36]. Similar to many bunyaviruses as well as TFV1, the replication of the Texas spiny softshell turtle virus in the brain of the suckling mice supports its neuroinvasive capacity. Unfortunately, the Texas spiny softshell turtle virus was never genetically characterized for comparison to TFV1 and the authors did not attempt to transmit the virus to turtles. Future investigation is needed to determine the route(s) of TFV1 transmission including the potential role of hematophagous insects that bite trionychid and emydid turtles when they are hauled out of the water, the role of ectoparasites (e.g., infected leeches) or endoparasites (spirorchid flukes), whether horizontal transmission occurs when adults congregate for mating, and/or whether vertical transmission occurs as has been reported for boa constrictors (*Boa constrictor*) infected with reptarenaviruses [37].

The index case of TFV1 infection was a softshell turtle submitted to the Bronson Animal Disease Diagnostic Laboratory (BADDL) as part of a disease episode on a Florida freshwater turtle farm in 2007. Unfortunately, additional details of this epizootic were not available, including confident identification of the host species. Although the scale of the Florida freshwater turtle industry has not been estimated like in other southern states [38], farms in Florida are currently rearing many native and exotic turtle species for the food and/or pet trades (P. Sapp pers. comm.). The detection of nearly identical strains of TFV1 from epizootics involving multiple distantly-related species of both managed and free-ranging freshwater turtles underscores the importance of farm biosecurity to prevent the introduction/escape of viruses like TFV1 onto/off farms through the introduction/escape of infected turtles, use/discharge of contaminated water sources (e.g., surface water), and through fomites. To some extent, the risk of moving infected free-ranging turtles onto farms has been mitigated by 2009 legislation that banned the collection of turtles from natural waterways for commercial purposes (e.g., slaughter, ranching) in Florida. This legislation was meant to protect Florida turtle species, including freshwater species like softshell turtles (*Apalone* spp.) and common snapping turtles (*Chelydra serpentina*), from being overharvested to meet the demand of Asian seafood markets in the US and abroad [2].

Since 2018, FWC and partners have investigated annual epizootics linked to TFV1 involving three free-ranging freshwater turtle species in multiple locations within Florida. These investigations confirmed TFV1 is not restricted to a single Florida watershed and that the disease typically occurs in adults during the late winter to spring months (late January through late May). Although preliminary, this temporal and age-related association may indicate TFV1 transmission and subsequent disease occurs when the immune system of adult turtles is impaired from overwintering and/or from the hormonal and physical stressors or elevated transmission probability associated with mating congregations in the spring. Study of future freshwater turtle epizootics will help better define the distribution of TFV1 across Florida and whether these events are preceded by environmental and/or anthropogenic stressors.

We do not know how long TFV1 may have been in wild turtle populations or its current distribution. Although there has been increased interest in diseases of free-ranging reptiles in recent decades, reporting and disease investigation efforts are dwarfed by those involving wildlife with economic, human health, or agricultural importance. Moreover, there is potential that this virus was previously missed as a result of concurrent secondary infections or parasitic lesions. It cannot be ruled out that the purported neuroinvasive peribunyavirus isolated from a Texas spiny softshell turtle in early 1970s represents the earliest detection of TFV1. In addition, the impact of the TFV1 epizootics on managed and free-ranging freshwater turtle populations in Florida cannot be determined from the data presented in this study. Fortunately, Florida populations of each of the three known susceptible species are considered secure [2] and all are listed as species of least concern by the IUCN Red List (https://www.iucnredlist.org/). However, future experimental challenge studies and continued surveillance efforts are needed to determine the host range of TFV1, and thus, any potential threat it poses to Florida’s rare, vulnerable, and endangered freshwater turtle species [2]. Given the history of anthropogenic movement of freshwater turtles within the US and abroad for trade, it is possible (if not likely) that TFV1 will be found in other areas and will become recognized as a significant chelonian pathogen of concern.

## Supporting information

S1 Fig Amino acid sequence alignment displaying conserved domains of the Turtle fraservirus 1 (2007 and 2020 strains) RNA-dependent RNA polymerase (highlighted in yellow) when compared to an assortment of negarnaviruses. Amino acid positions with a threshold of conservation >85% are highlighted in grey.
(TIF)

S2 Fig Amino acid sequence of the Turtle fraservirus 1 (2007 and 2020 strains) putative zinc-binding matrix protein. Potential metal binding motif (Cx3C…Hx3H) underlined and key amino acid residues highlighted in yellow.
(TIF)

## References

[1] www.floridamuseum.ufl.edu [Internet]. Gainesville, FL: Checklist of Florida Turtles [cited 2022 February 15]. Available from: https://www.floridamuseum.ufl.edu/discover-herps/florida-amphibians-reptiles/turtles/.

[2] Krysko KL, Enge KM, Moler PE. Amphibians and Reptiles of Florida. Gainesville: University of Florida Press; 2019.

[3] Buhlmann KA, Akre TS, Iverson JB, Karapatakis D, Mittermeier RA, Georges A, et al. A global analysis of tortoise and freshwater turtle distributions with identification of priority conservation areas. Chelonian Conserv and Biol. 2009 Dec 1;8(2):116–149. 10.2744/CCB-0774.1.

[4] Turtle Conservation Fund. A Global Action Plan for Conservation of Tortoises and Freshwater Turtles: Strategy and Funding Prospectus 2002–2007. Washington, DC: Conservation International and Chelonian Research Foundation; 2002.

[5] Vié JC, Hilton-Taylor C, Stuart SN, editors. Wildlife in a Changing World–An Analysis of the 2008 IUCN RedList of Threatened Species. Gland, Switzerland: International Union for Conservation of Nature; 2009.

[6] Heppell SS. Application of life-history theory and population model analysis to turtle conservation. Copeia. 1998 May;2(2):367–375.

[7] Brooks RJ, Brown GP, Galbraith DA. Effects of a sudden increase in natural mortality of adults on a population of the common snapping turtle (Chelydra serpentina). Canadian Journal of zoology. 1991 May 1;69(5):1314–1320. 10.2307/1447430.

[8] Harvell CD, Kim K, Burkholder JM, Colwell RR, Epstein PR, Grimes DJ, et al. Emerging marine diseases—climate links and anthropogenic factors. Science. 1999 Sep 3;285(5433):1505–10. doi: 10.1126/science.285.5433.1505 . 10498537

[9] Daszak P, Cunningham AA, Hyatt AD. Emerging infectious diseases of wildlife—threats to biodiversity and human health. Science. 2000 Jan 21;287(5452):443–9. Erratum in: Science 2000 Mar 10;287(5459):1756. doi: 10.1126/science.287.5452.443 . 10642539

[10] Gibbons PM, Steffes ZJ. Emerging infectious diseases of chelonians. Vet Clin North Am Exot Anim Pract. 2013 May;16(2):303–17. Epub 2013 Mar 15. doi: 10.1016/j.cvex.2013.02.004 . 23642864

[11] Jacobson ER, Gaskin JM, Wahlquist H. Herpesvirus-like infection in map turtles. J Am Vet Med Assoc. 1982 Dec 1;181(11):1322–1324. 6294034

[12] Ariel E. Viruses in reptiles. Vet Res. 2011 Sep 21;42(1):100. doi: 10.1186/1297-9716-42-100 ; PMCID: PMC3188478. 21933449 PMC3188478

[13] Marschang RE. Viruses infecting reptiles. Viruses. 2011 Nov;3(11):2087–126. Epub 2011 Nov 1. doi: 10.3390/v3112087 ; PMCID: PMC3230843. 22163336 PMC3230843

[14] Maes P, Alkhovsky SV, Bào Y, Beer M, Birkhead M, Briese T, et al. Taxonomy of the family Arenaviridae and the order Bunyavirales: update 2018. Arch Virol. 2018 Aug;163(8):2295–2310. Epub 2018 Apr 21. doi: 10.1007/s00705-018-3843-5 . 29680923

[15] Tatineni S, McMechan AJ, Wosula EN, Wegulo SN, Graybosch RA, French R, et al. An eriophyid mite-transmitted plant virus contains eight genomic RNA segments with unusual heterogeneity in the nucleocapsid protein. J Virol. 2014 Oct;88(20):11834–45. doi: 10.1128/JVI.01901-14 Epub 2014 Aug 6. Erratum in: J Virol. 2015 Jul;89(14):7443. ; PMCID: PMC4178757. 25100845 PMC4178757

[16] Schindelin J, Rueden CT, Hiner MC, Eliceiri KW. The ImageJ ecosystem: An open platform for biomedical image analysis. Mol Reprod Dev. 2015 Jul-Aug;82(7–8):518–29. Epub 2015 Jul 7. doi: 10.1002/mrd.22489 ; PMCID: PMC5428984. 26153368 PMC5428984

[17] Bankevich A, Nurk S, Antipov D, Gurevich AA, Dvorkin M, Kulikov AS, et al. SPAdes: a new genome assembly algorithm and its applications to single-cell sequencing. J Comput Biol. 2012 May;19(5):455–77. Epub 2012 Apr 16. doi: 10.1089/cmb.2012.0021 ; PMCID: PMC3342519. 22506599 PMC3342519

[18] Besemer J, Lomsadze A, Borodovsky M. GeneMarkS: a self-training method for prediction of gene starts in microbial genomes. Implications for finding sequence motifs in regulatory regions. Nucleic Acids Res. 2001 Jun 15;29(12):2607–2618. doi: 10.1093/nar/29.12.2607 ; PMCID: PMC55746. 11410670 PMC55746

[19] Zimmermann L, Stephens A, Nam SZ, Rau D, Kübler J, Lozajic M, et al. A Completely Reimplemented MPI Bioinformatics Toolkit with a New HHpred Server at its Core. J Mol Biol. 2018 Jul 20;430(15):2237–2243. Epub 2017 Dec 16. doi: 10.1016/j.jmb.2017.12.007 . 29258817

[20] Wolf YI, Kazlauskas D, Iranzo J, Lucía-Sanz A, Kuhn JH, Krupovic M, et al. Origins and Evolution of the Global RNA Virome. mBio. 2018 Nov 27;9(6):e02329–18. doi: 10.1128/mBio.02329-18 ; PMCID: PMC6282212. 30482837 PMC6282212

[21] Wolf YI, Silas S, Wang Y, Wu S, Bocek M, Kazlauskas D, et al. Doubling of the known set of RNA viruses by metagenomic analysis of an aquatic virome. Nat Microbiol. 2020 Oct;5(10):1262–1270. Epub 2020 Jul 20. doi: 10.1038/s41564-020-0755-4 ; PMCID: PMC7508674. 32690954 PMC7508674

[22] Edgar RC. MUSCLE v5 enables improved estimates of phylogenetic tree confidence by ensemble bootstrapping. bioRxiv. 2021 Jun. 10.1101/2021.06.20.449169.

[23] Esterman ES, Wolf YI, Kogay R, Koonin EV, Zhaxybayeva O. Evolution of DNA packaging in gene transfer agents. Virus Evol. 2021 Feb 19;7(1):veab015. doi: 10.1093/ve/veab015 PMCID: PMC7947584. 33732503 PMC7947584

[24] Nguyen LT, Schmidt HA, von Haeseler A, Minh BQ. IQ-TREE: a fast and effective stochastic algorithm for estimating maximum-likelihood phylogenies. Mol Biol Evol. 2015 Jan;32(1):268–74. Epub 2014 Nov 3. doi: 10.1093/molbev/msu300 ; PMCID: PMC4271533. 25371430 PMC4271533

[25] Wang F, Flanagan J, Su N, Wang LC, Bui S, Nielson A, et al. RNAscope: a novel in situ RNA analysis platform for formalin-fixed, paraffin-embedded tissues. J Mol Diagn. 2012 Jan;14(1):22–9. doi: 10.1016/j.jmoldx.2011.08.002 ; PMCID: PMC3338343. 22166544 PMC3338343

[26] Anderson CM, Zhang B, Miller M, Butko E, Wu X, Laver T, et al. Fully Automated RNAscope In Situ Hybridization Assays for Formalin-Fixed Paraffin-Embedded Cells and Tissues. J Cell Biochem. 2016 Oct;117(10):2201–8. Epub 2016 Jun 6. doi: 10.1002/jcb.25606 ; PMCID: PMC5132049. 27191821 PMC5132049

[27] Keller KA, Guzman DS, Paul-Murphy J, Byrne BA, Owens SD, Kass PH, et al. Hematologic and plasma biochemical values of free-ranging western pond turtles (Emys marmorata) with comparison to a captive population. J Herpet Med Surg. 2012 Sep 1;22(3–4):99–106. 10.5818/1529-9651-22.3.99.

[28] Hierweger MM, Koch MC, Rupp M, Maes P, Di Paola N, Bruggmann R, et al. Novel Filoviruses, Hantavirus, and Rhabdovirus in Freshwater Fish, Switzerland, 2017. Emerg Infect Dis. 2021 Dec;27(12):3082–3091. doi: 10.3201/eid2712.210491 ; PMCID: PMC8632185. 34808081 PMC8632185

[29] Rastgou M, Habibi MK, Izadpanah K, Masenga V, Milne RG, Wolf YI, et al. Molecular characterization of the plant virus genus Ourmiavirus and evidence of inter-kingdom reassortment of viral genome segments as its possible route of origin. J Gen Virol. 2009 Oct;90(Pt 10):2525–2535. Epub 2009 Jun 17. doi: 10.1099/vir.0.013086-0 ; PMCID: PMC4091139. 19535502 PMC4091139

[30] Stenglein MD, Sanders C, Kistler AL, Ruby JG, Franco JY, Reavill DR, et al. Identification, characterization, and in vitro culture of highly divergent arenaviruses from boa constrictors and annulated tree boas: candidate etiological agents for snake inclusion body disease. mBio. 2012 Aug 14;3(4):e00180–12. doi: 10.1128/mBio.00180-12 ; PMCID: PMC3419519. 22893382 PMC3419519

[31] Bang FB. Transmissible Disease, Probably Viral in Origin, Affecting the Amebocytes of the European Shore Crab, Carcinus maenas. Infect Immun. 1971 Apr;3(4):617–23. doi: 10.1128/iai.3.4.617-623.1971 ; PMCID: PMC416205. 16558026 PMC416205

[32] Bang FB. Pathogenesis and autointerference in a virus disease of crabs. Infect Immun. 1974 Jun;9(6):1057–61. PMCID: PMC414932. doi: 10.1128/iai.9.6.1057-1061.1974 4857421 PMC414932

[33] Bang FB. Crustacean disease responses. In: Provenzano AJ, editor. The biology of Crustacea. New York: Academic Press; 1983. pp. 114–153.

[34] Sakuna K, Elliman J, Tzamouzaki A, Owens L. A novel virus (order Bunyavirales) from stressed redclaw crayfish (Cherax quadricarinatus) from farms in northern Australia. Virus Res. 2018 May 2;250:7–12. Epub 2018 Mar 21. doi: 10.1016/j.virusres.2018.03.012 . 29574101

[35] Waltzek TB, Subramaniam K, Leis E, Katona R, Fan Ng TF, Delwart E, et al. Characterization of a peribunyavirus isolated from largemouth bass (Micropterus salmoides). Virus Res. 2019 Nov;273:197761. Epub 2019 Sep 17. doi: 10.1016/j.virusres.2019.197761 . 31539558

[36] Hoff G, Trainer DO. Arboviruses in Reptiles: Isolation of a Bunyamwera Group Virus from a Naturally Infected Turtle. J Herpetol. 1973;7(2):55–62. 10.2307/1563201.

[37] Keller S, Hetzel U, Sironen T, Korzyukov Y, Vapalahti O, Kipar A, et al. Co-infecting Reptarenaviruses Can Be Vertically Transmitted in Boa Constrictor. PLoS Pathog. 2017 Jan 23;13(1):e1006179. doi: 10.1371/journal.ppat.1006179 ; PMCID: PMC5289648. 28114434 PMC5289648

[38] Hughes DW. The Contribution of the Pet Turtle Industry to the Louisiana Economy. Aquac Econ Manag. 1999;3(3):205–214. 10.1080/13657309909380247.

