# Supplementary figures and images for: A novel group of negative-sense RNA viruses associated with epizootics in managed and free-ranging freshwater turtles in Florida, USA
Source: PLoS Pathog. 2022 Mar 11;18(3):e1010258. doi: 10.1371/journal.ppat.1010258 (PMC8916662; doi:10.1371/journal.ppat.1010258)

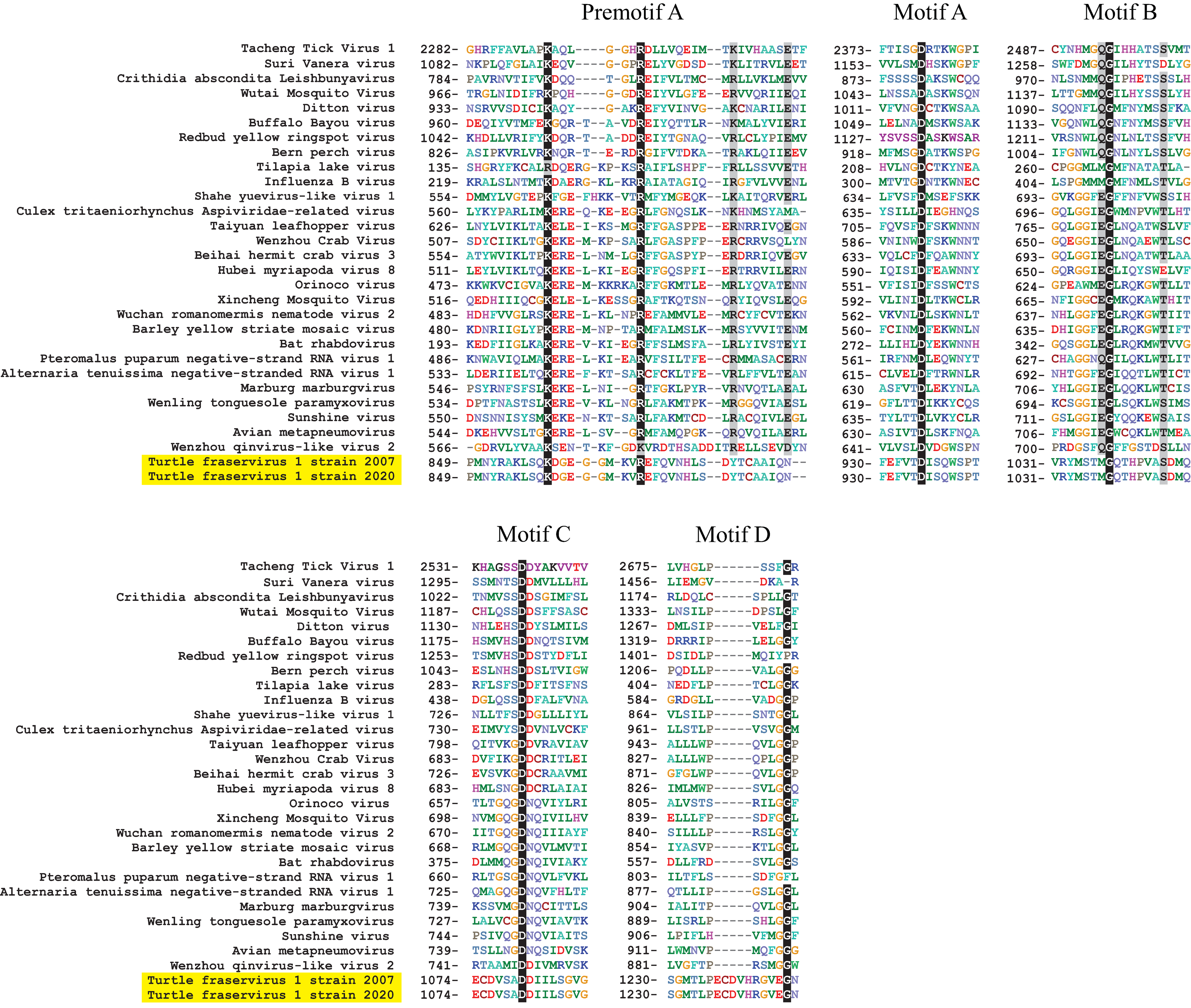

Supplement: S1 Fig — Amino acid positions with a threshold of conservation >85% are highlighted in grey. (TIF) [file ppat.1010258.s001.tif]

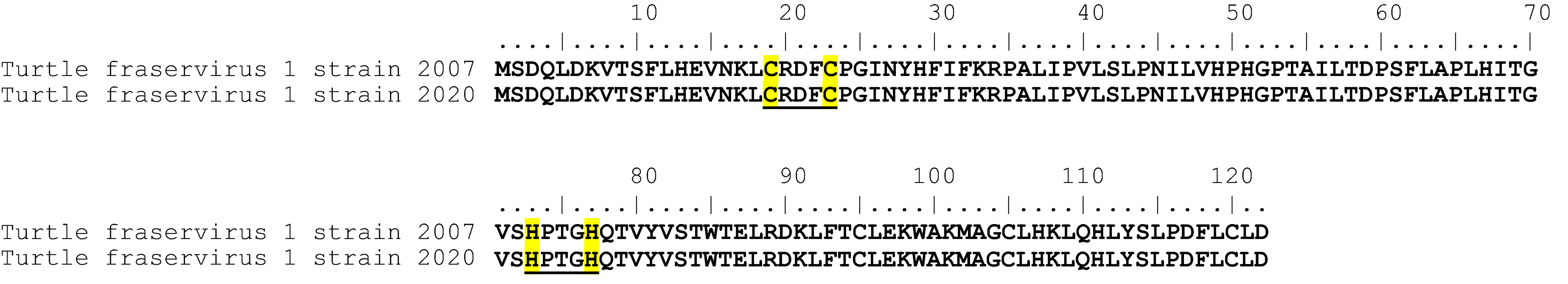

Supplement: S2 Fig — Potential metal binding motif (Cx3C…Hx3H) underlined and key amino acid residues highlighted in yellow. (TIF) [file ppat.1010258.s002.tif]
